# Mechanical Properties and Energy Absorption Characteristics of Ring Lattice Sandwich Structures Under Compressive Load

**DOI:** 10.3390/ma19163520

**Published:** 2026-08-19

**Authors:** Wenkang Wang, Xinsheng Jiang, Yu Liao, Zhenhua Tian

**Affiliations:** 1 Joint Logistics Support Force University of Engineering, Chongqing 401311, China; jxs_dy@163.com (X.J.); t814822690@163.com (Z.T.); 2 Defense Engineering Institute, Academy of Military Science, PLA, Luoyang 471000, China; 3 Air Force Engineering University, Xi’an 710038, China; creast891230@163.com

**Keywords:** ring lattice sandwich structure, interlocking and assembly, quasi-static compression, SHPB test, mechanical properties, energy absorption

## Abstract

To enhance critical infrastructure protection against low-cost UAV impacts, this study proposes a novel ring lattice sandwich structure (RLSS) fabricated via an economical interlocking-assembly-brazing method. Its quasi-static compressive behavior is systematically investigated through experiments, numerical simulations, and theoretical analysis. Theoretical models for relative density and initial yield stress are validated against experiments, with errors of 7.1% and 6.6%, respectively. Quasi-static tests show that the one-layer RLSS exhibits a specific energy absorption (SEA) of 8.67 J/g, while the two-layer structure drops to 5.66 J/g due to inter-layer torsional instability. SHPB impact tests at strain rates of 750–1369 s^−1^ demonstrate a pronounced strain-rate effect, with dynamic increase factors ranging from 1.14 to 1.43. Numerical simulations accurately reproduce the experimental deformation modes and reveal that multi-layer (2–5 layers) RLSSs reduce SEA by 46.9% compared with the one-layer simulated value of 9.43 J/g. Adding a 0.3-mm inner panel in simulations restores the crushing mode and raises the SEA of the two-layer structure to 7.19 J/g, surpassing the non-panel counterpart (6.03 J/g). Hybrid core configurations provide additional advantages: Mode I (ring–pyramid with inner panel) enhances total energy absorption with a limited ring-layer count, while Mode II (alternating layers) achieves minimal plateau stress fluctuation (PSF = 0.09). These findings confirm that the proposed RLSS, especially when optimized with thin inner panels or hybrid designs, offers great potential as protective cladding against impact and blast threats.

## 1. Introduction

In recent regional conflicts, the deployment of low-cost Unmanned Aerial Vehicles (UAVs) against oil production and storage infrastructure has emerged as a potent asymmetric threat. Given the inherent structural vulnerabilities of these facilities, they are highly susceptible to ignition and catastrophic explosion upon UAV impact. Such incidents carry the risk of triggering cascading domino effects across the entire production and storage complex [1]. Consequently, enhancing the resilience of these critical facilities against UAV-induced damage constitutes an urgent and pivotal research topic.

A promising strategy to improve the explosion resistance of oil production and storage facilities involves encasing them in advanced protective cladding. In the realm of traditional explosion protection, methods such as polyurea coatings [2], high-performance fiber materials [3], and lattice structures [4] are commonly employed. While polyurea coatings and high-performance fiber materials offer ease of application, they are prone to softening and failure under the high-temperature conditions associated with combustion explosions, resulting in limited improvement in blast protection performance [5,6]. In contrast, lattice structures feature low density, high specific stiffness, high specific strength, and exceptional designability, making them widely applicable in explosion-resistant protective systems [7,8,9]. Lattice structures can be categorized into two-dimensional (2D) and three-dimensional (3D) configurations. 2D lattice structures primarily refer to grid structures formed by the periodic arrangement of polygons, such as honeycomb [10] and corrugated lattice structures [11]. 3D lattice structures, however, consist of spatial truss systems formed by the repeated arrangement of micro-elements (e.g., rods and plates) according to specific topological rules [12], including tetrahedral, pyramidal, and 3D-Kagome architectures, etc. Compared to 2D lattice structures, 3D lattice structures offer superior design flexibility, allowing for better conformity to the curved surfaces typical of energy infrastructure. Furthermore, the interconnected internal voids of 3D lattice structures provide a versatile platform for integrating additional fire and explosion suppression functionalities [13].

The energy absorption capacity of lattice structures is a primary focus of current research, typically evaluated based on the energy dissipated during compression [14]. This capacity is significantly influenced by geometric configurations, with different unit cell types exhibiting distinct deformation modes and energy absorption efficiencies [15]. In traditional 3D truss lattice structures, energy dissipation is primarily dominated by rod buckling, a mechanism that frequently results in undesirable high initial peak forces and oscillating load-bearing capacities [16]. Replacing straight struts with curved (arched) struts alters the energy dissipation mode, yielding a more stable reaction force output during out-of-plane compression [17]. Additionally, fabrication processes play a critical role in determining the energy absorption performance of lattice structures. Current typical fabrication methods include stamping and folding [18], interlocking and assembly [19], and additive manufacturing [20], etc. While stamping and folding is simple and suitable for mass production, it struggles to produce complex configurations. Additive manufacturing enables the fabrication of intricate geometries but is constrained by long lead times and high costs, limiting its scalability. The interlocking and assembly method, which involves assembling cut plate components, offers a balanced solution, enabling the batch production of lattice structures with complex unit cells while maintaining cost-effectiveness.

Currently, few studies have focused on the arch lattice sandwich structures prepared by the interlocking and assembly method, which restricts their application in the field of explosion-resistant protection. In this paper, a ring lattice sandwich structure (RLSS) was proposed prepared by the interlocking-assembly-brazing method. The mechanical properties and energy absorption characteristics of the structure under compression load were analyzed through experiments, numerical simulations and theoretical analysis. Based on this, the influences of different parameters on the mechanical properties and energy absorption of the RLSSs were studied. The relevant research lays a solid foundation for the application of the RLSSs.

## 2. Materials and Methods

### 2.1. Fabrication of Lattice Sandwich Structure Samples

**Figure 1:**
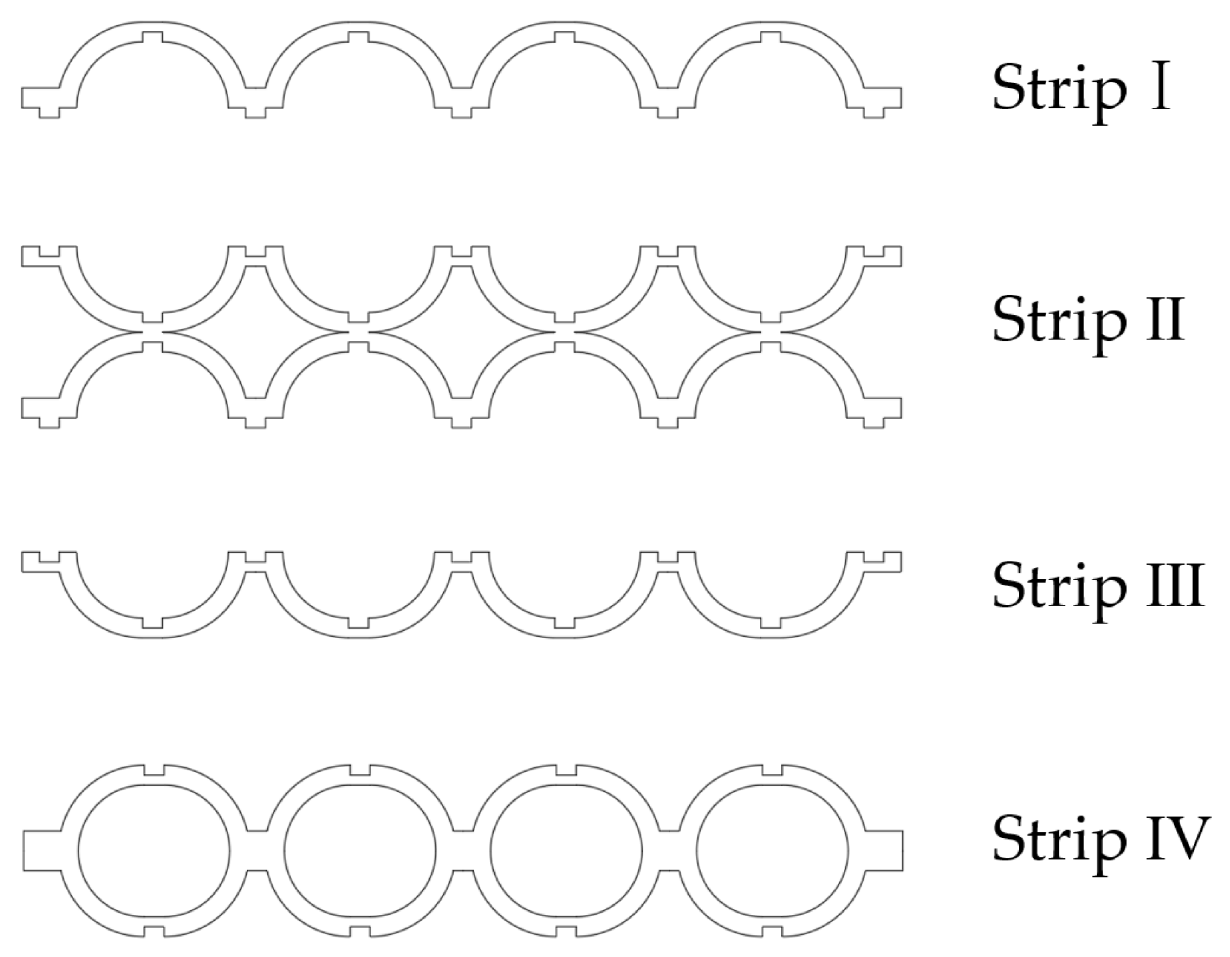
Basic interlocking assembly unit of ring lattice core.

Based on the topological analysis method, the spatial topological relationship of the RLSS was disassembled. The core layer of the RLSS was disassembled into three (one layer) or four (two layers and above) basic interlocking strips, as shown in Figure 1. The interlocking strips were cut with convex grooves or concave grooves, which are used for mutual engagement and interlocking of the strips. The width and depth of the cuts are both half the thickness of the interlocking assembly strips.

**Figure 2:**
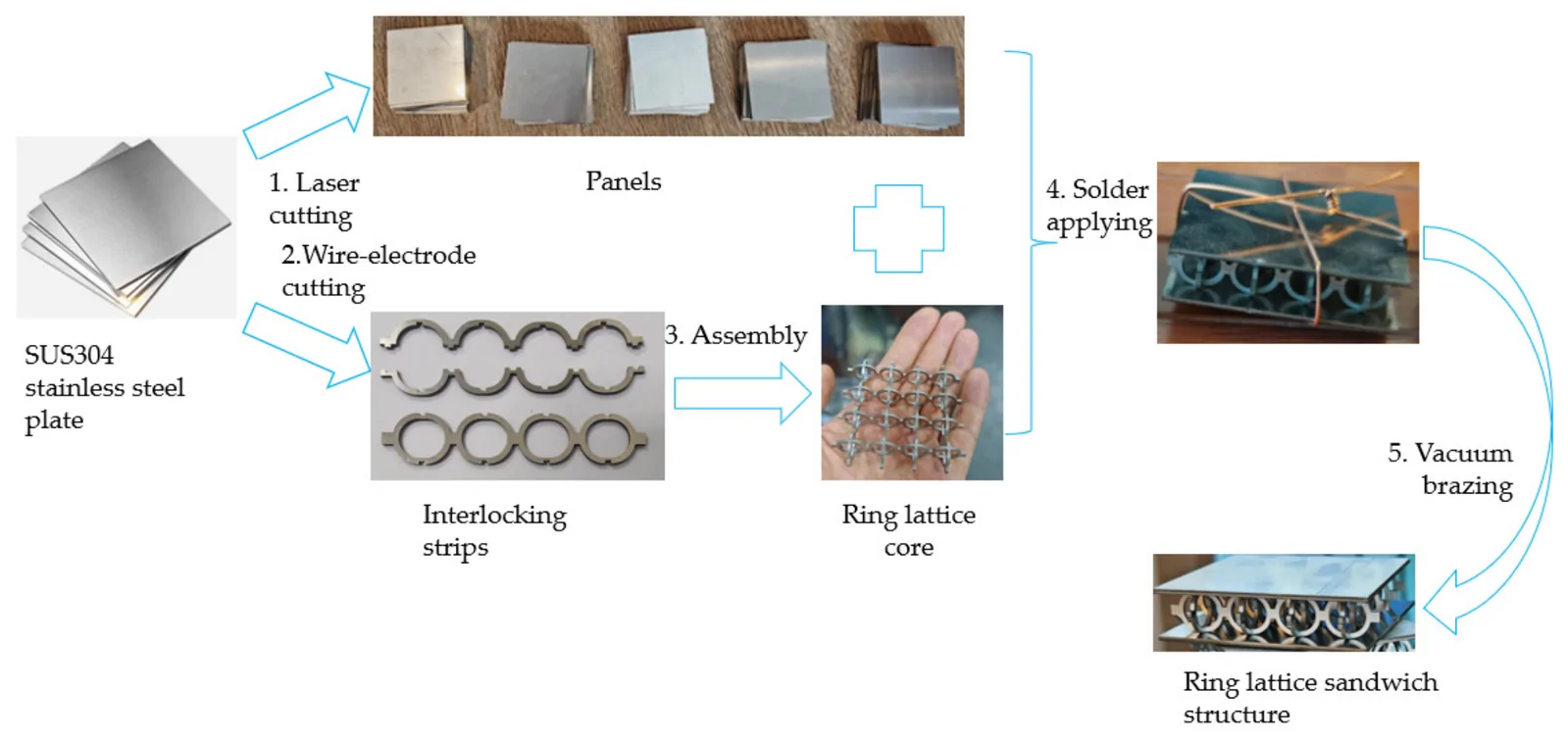
Fabrication process of the ring lattice sandwich structure.

The preparation of the RLSS test samples was carried out through the following four processes, as shown in Figure 2. Firstly, square panels of 67.0 mm × 67.0 mm were cut from a 1.5 mm thick SUS304 stainless steel plate using laser cutting technology. Secondly, interlocking strips were cut from the same 1.5-mm-thick SUS304 stainless steel plate using slow-moving wire-electrode cutting technology. Due to the small size of the interlocking assembly strips, laser cutting technology may easily cause deformation of the assembly strips, so slow-moving wire-electrode cutting technology was adopted here. Thirdly, these interlocking strips were interlocked with each other to form a ring lattice core. For the one-layer RLSS, it was assembled from three types of interlocking strips: Strip I, Strip III, and Strip IV. Strip III was placed at the bottom in the meridional direction, then Strip IV was placed in the latitudinal direction on the grooves of Strip III, and finally Strip I was placed in the meridional direction on the upper grooves of Strip IV. For the multi-layer ring lattice sandwich structure, it was assembled from four types of interlocking strips: Strip I, Strip II, Strip III, and Strip IV. The basic assembly process is similar to that of the one-layer structure, except that Strip II is used for the meridional interlocking strips in the middle layer(s). Fourthly, a kind of BNi-2 solder was evenly applied to the interlocking grooves of the interlocking assembly strips and the contact areas between the interlocking strips and the panel. Finally, the core was placed between two SUS304 stainless steel panels, with a metal block placed on the upper panel to ensure sufficient contact between the panels and the core. The assembly structures were placed in a high-temperature vacuum brazing furnace, gradually heated to 1050 °C, and maintained at 2 × 10^−2^ Pa for about 10 min. Afterwards, the vacuum furnace was allowed to cool naturally to room temperature.

**Figure 3:**
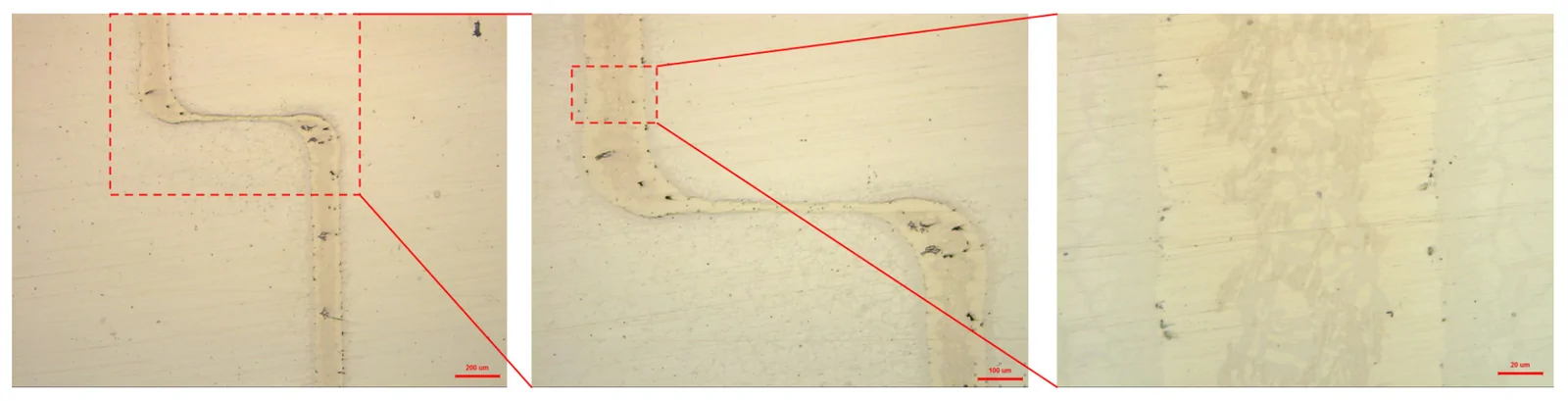
Microscopic morphology of the weld joint of RLSS.

Samples were taken from the joint of the interlocking strips using wire electrical discharge machining. After surface preparation, metallographic analysis of the vacuum-brazed seam of the RLSS structure was conducted via Olympus GX51 optical microscopy (Olympus Corporation, Inc., Nagano, Japan), as shown in Figure 3. The metallographic micrographs indicate that the brazing filler metal is well bonded with the surrounding base metal, and the brazed joint exhibits good quality with no apparent defects.

### 2.2. Quasi-Static Tensile Tests of Furnace-Accompanied Base Material

To ensure the accuracy of theoretical analysis and numerical simulation, a furnace-accompanied base material was set up during the fabrication of the ring lattice sandwich structure. Quasi-static tensile tests were conducted on the furnace-accompanied base material to obtain its basic mechanical property parameters.

According to “Metallic Materials—Tensile Testing—Part 1: Method of Test at Room Temperature” (GB/T228.1-2021 [21]), dumbbell-shaped quasi-static tensile specimens were cut from the heat-treated test base material using a laser cutting machine. A CBW-10T universal testing machine (Chongqing Chongbiao Instruments Co., Ltd., Chongqing, China) was used for uniaxial tensile testing, with the compression rate set to 2.5 mm/min, to obtain the engineering stress–strain curve of the specimens under quasi-static tensile conditions. The engineering stress–strain curve obtained from the testing machine is derived by dividing the applied force and deformation by the original dimensions of the specimen. However, after the elastic phase, the material enters the plastic deformation phase, and its cross-sectional area decreases as the specimen elongates, resulting in the “necking” phenomenon. Consequently, the true stress of the specimen is significantly higher than the engineering stress, and the engineering stress–strain curve cannot accurately reflect the strain hardening behavior during material tensile deformation. Therefore, to deeply analyze the rheological behavior of the material, it is necessary to convert the engineering stress–strain curve obtained from the testing machine into a true stress–strain curve based on the assumption of material incompressibility. Based on the assumption of material incompressibility, the conversion relationship between true stress–strain and engineering stress–strain can be expressed as:

(1) $$\sigma_{T}(t)=[1+\varepsilon (t)]\sigma (t)$$

(2) $$\varepsilon_{T}(t)=\ln[1+\varepsilon (t)]$$

where $\sigma_{T}(t)$ is the true stress at time *t*, $\varepsilon_{T}(t)$ is the true strain at time *t*, σ(t) is the engineering stress at time *t*, and ε(t) is the engineering strain at time *t*.

**Figure 4:**
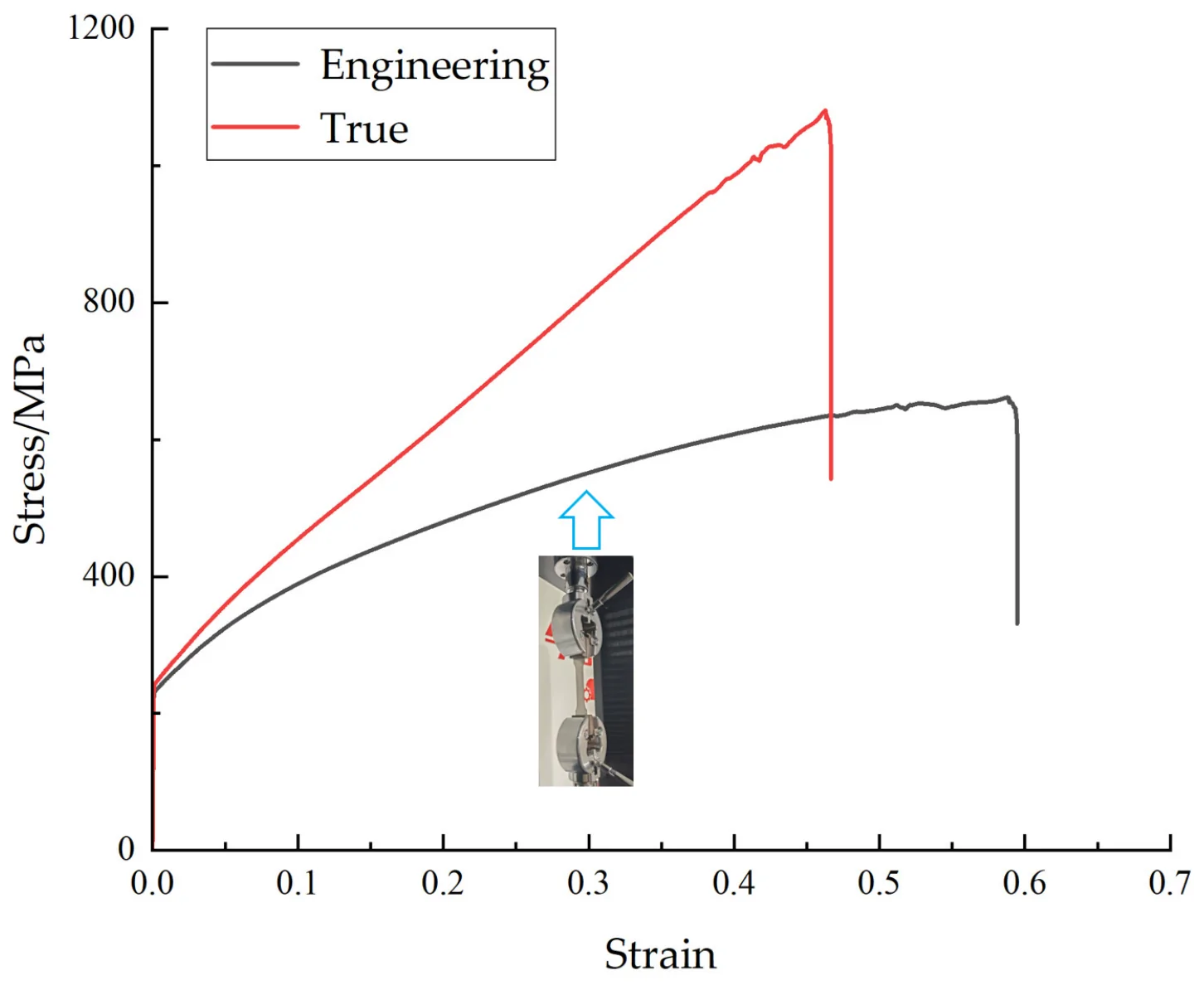
Engineering and true stress–strain curves of the furnace-accompanied base material.

The engineering and true stress–strain curves of the furnace-accompanied base material is as shown in Figure 4, and the key mechanical parameters are listed in Table 1.

### 2.3. Quasi-Static Compressive Tests of the Ring Lattice Sandwich Structures

**Figure 5:**
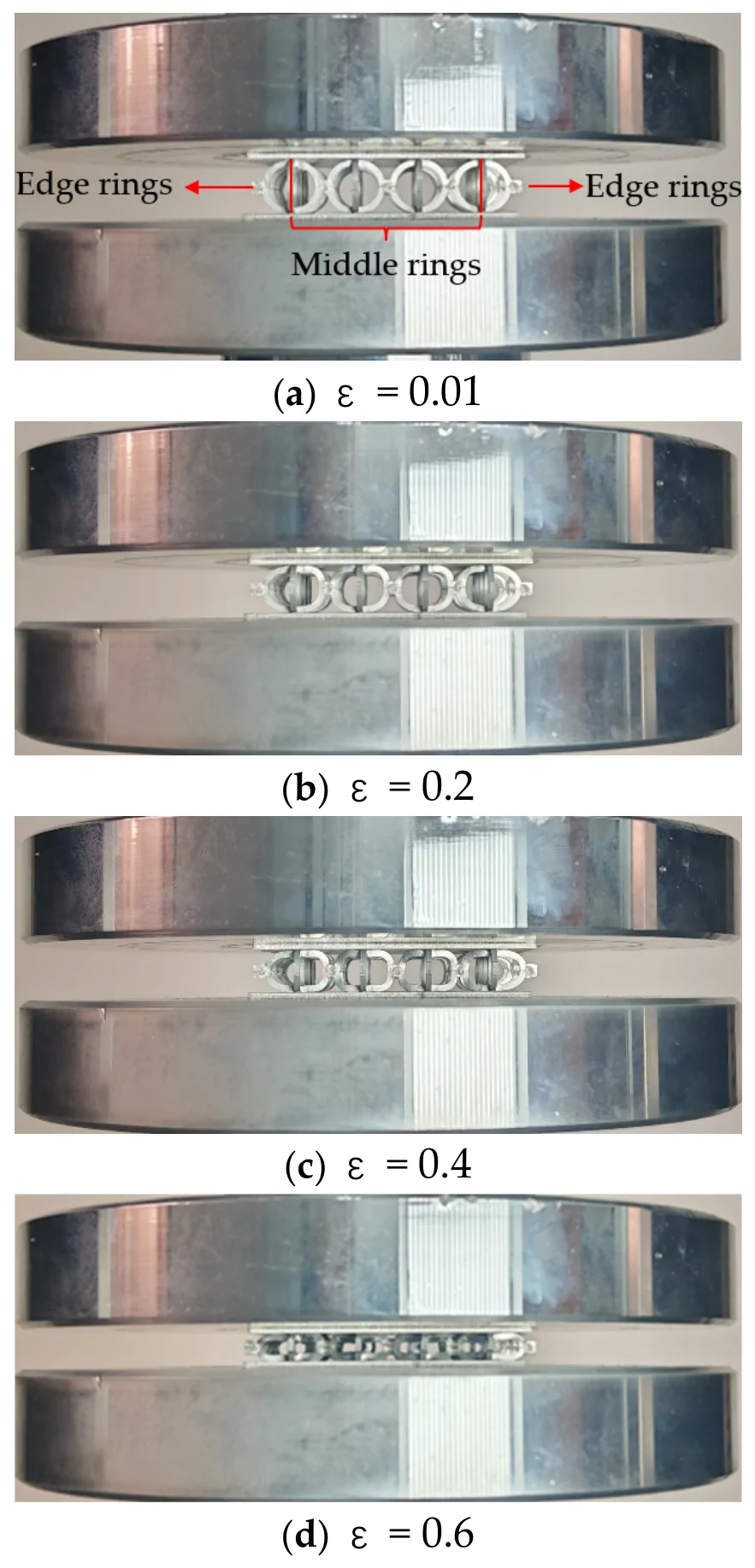
Quasi-static compression process of RLSS (4 × 4 × 1).

**Figure 6:**
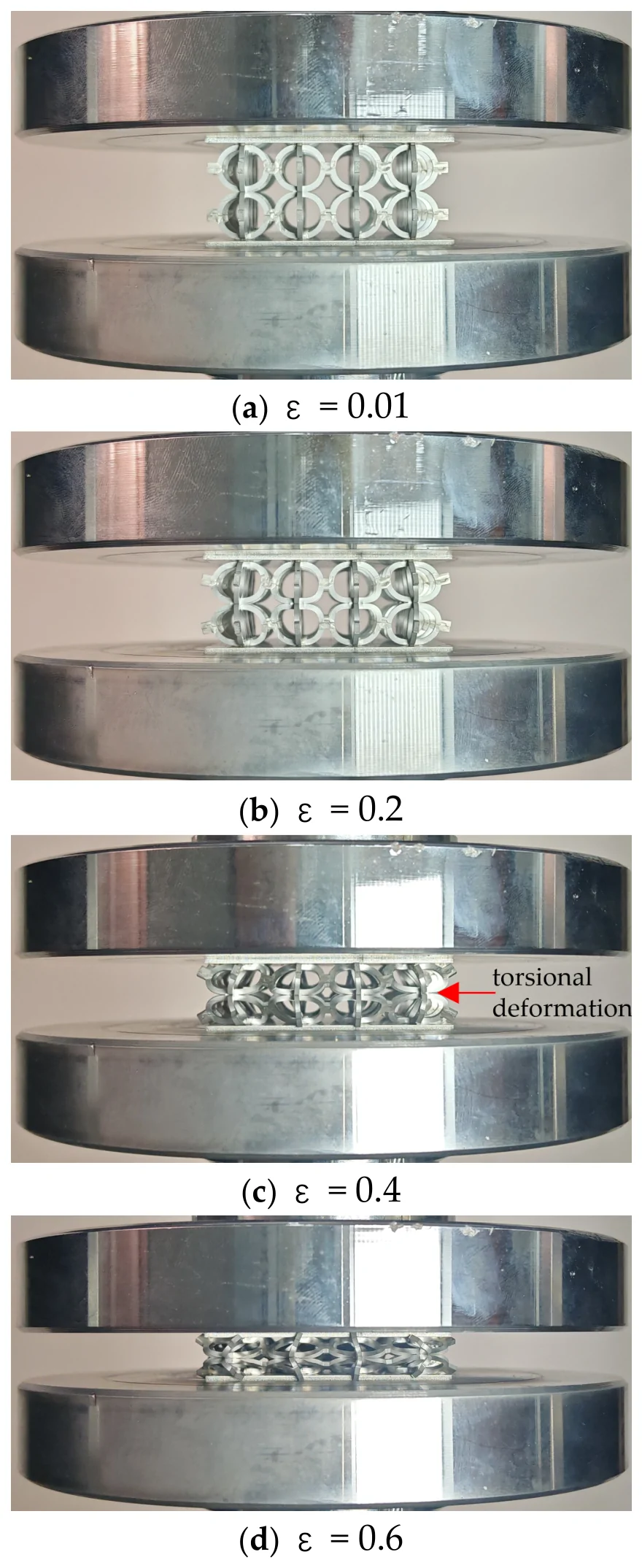
Quasi-static compression process of RLSS (4 × 4 × 2).

**Figure 7:**
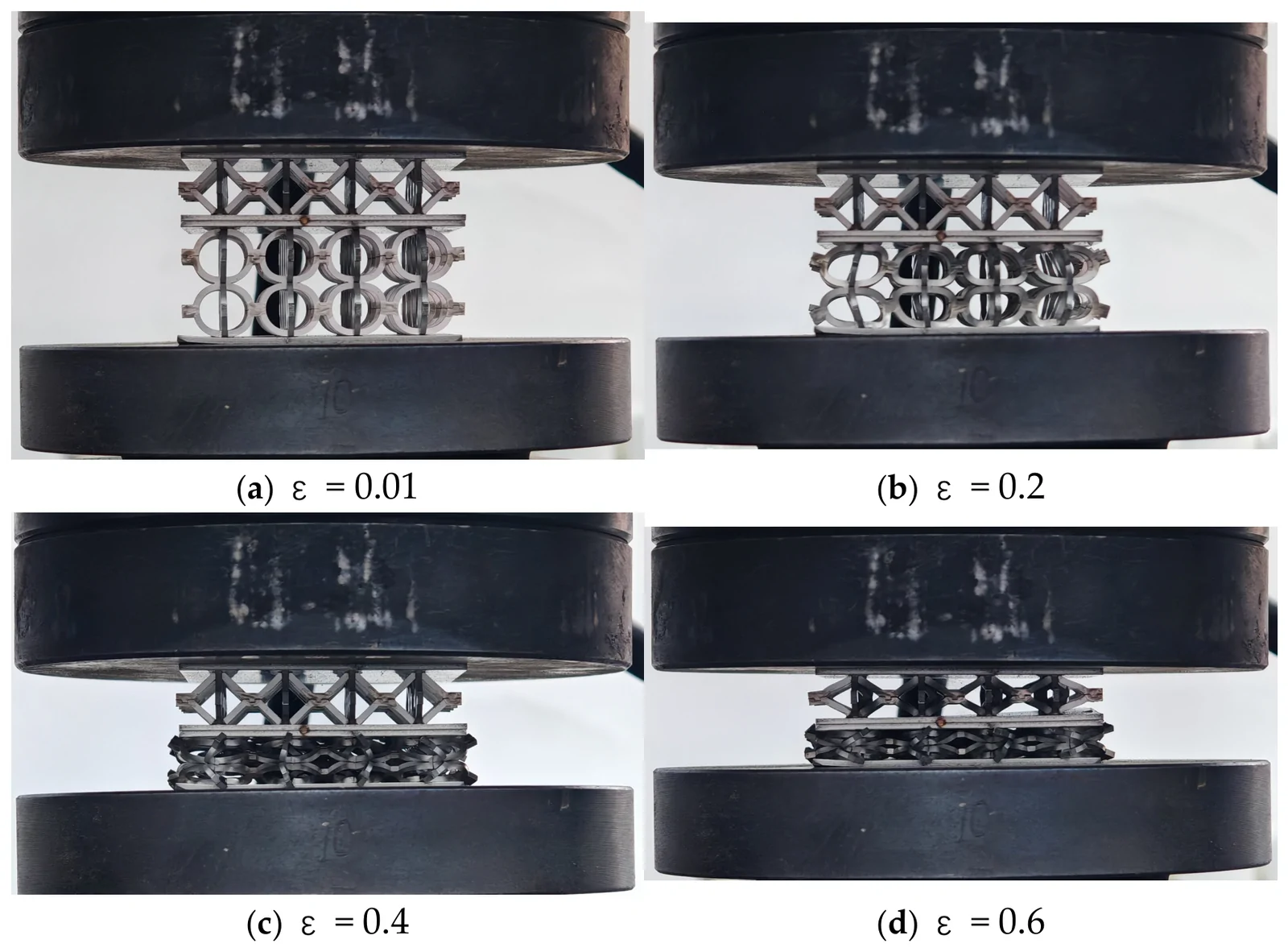
Quasi-static compression process of hybrid core PRLSS.

To analyze the mechanical properties and energy absorption characteristics of the RLSS under quasi-static load, quasi-static compressive tests were conducted on one-layer RLSS, two-layer RLSS and hybrid core PRLSS (pyramid–ring lattice sandwich structure). The hybrid core PRLSS was consisting of a one-layer PLSS (pyramid lattice sandwich structure) and a two-layer RLSS with an inner panel. The compressive tests were carried out on a universal testing machine. Specifically, a CBW-10T machine was used for the one-layer and two-layer RLSS, whereas a WAW-60T machine (Tianshui Hongshan Testing Machine Co., Ltd., Tianshui, China) was employed for the PRLSS. For all tests, the loading speed was fixed at 2.5 mm/min. The ring lattice structure is simplified and recorded as RLSS (m × n × k), where m, n, and k represent the number of rings in the meridional, latitudinal, and height directions, respectively. The core dimensions of RLSS (4 × 4 × 1) used in the tests were 67.0 mm × 67.0 mm × 13.0 mm, and those of RLSS (4 × 4 × 2) were 67.0 mm × 67.0 mm × 26.0 mm. To analyze the energy dissipation mechanism of the RLSS, PLSS, and PRLSS, the deformation mode during compression was recorded by a camera, as shown in Figure 5, Figure 6 and Figure 7. It can be seen from Figure 5 that as the upper panel descends, the rings in the core layer undergo compressive deformation, exhibiting two different crushing modes due to different constraints. For middle rings, the contact areas with the panels and the nodal interlocking positions transition from arcs to straight segments, and the individual ring transforms from a circle to a rounded rectangle. As deformation increases, the length of the straight segments increases while the length of the arc segments decreases. For edge rings, only the contact areas with the panels transition from arcs to straight segments, with the straight segment length increasing and the arc segment length decreasing as deformation increases. Comparing Figure 5, Figure 6 and Figure 7, it can be seen that due to the weakening of inter-layer nodal constraints, the deformation mode of the two-layer RLSS core changes significantly. In addition to vertical crushing deformation, the rings in the core also exhibit unstable torsional deformation at the contact points between the upper and lower core layers. As shown in Figure 7, the lower two-layer ring core collapsed first, exhibiting a deformation mode similar to that of the standalone two-layer ring core. After the ring core was fully crushed, the upper one-layer pyramidal core then underwent crushing and dissipated energy.

**Figure 8:**
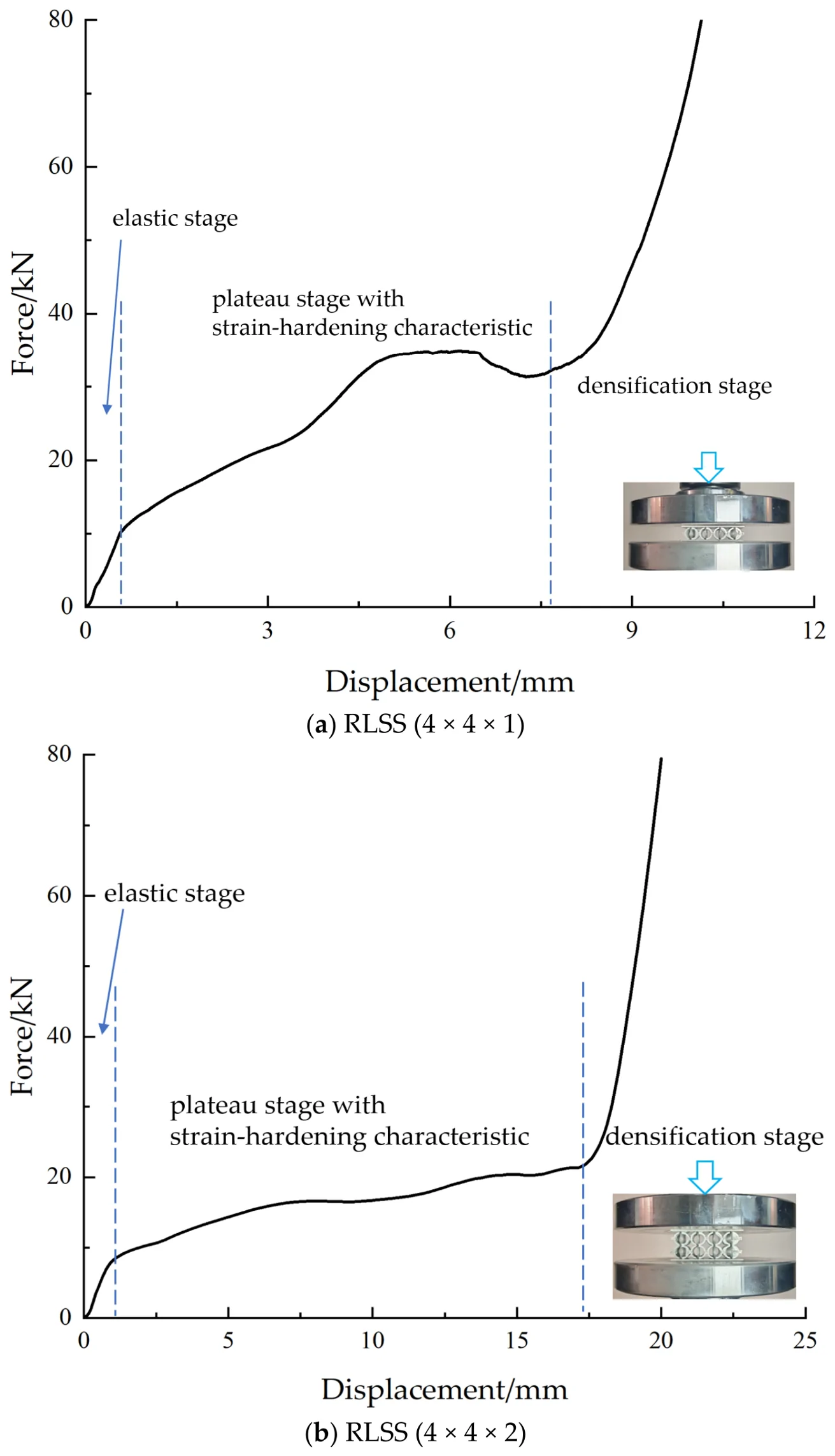

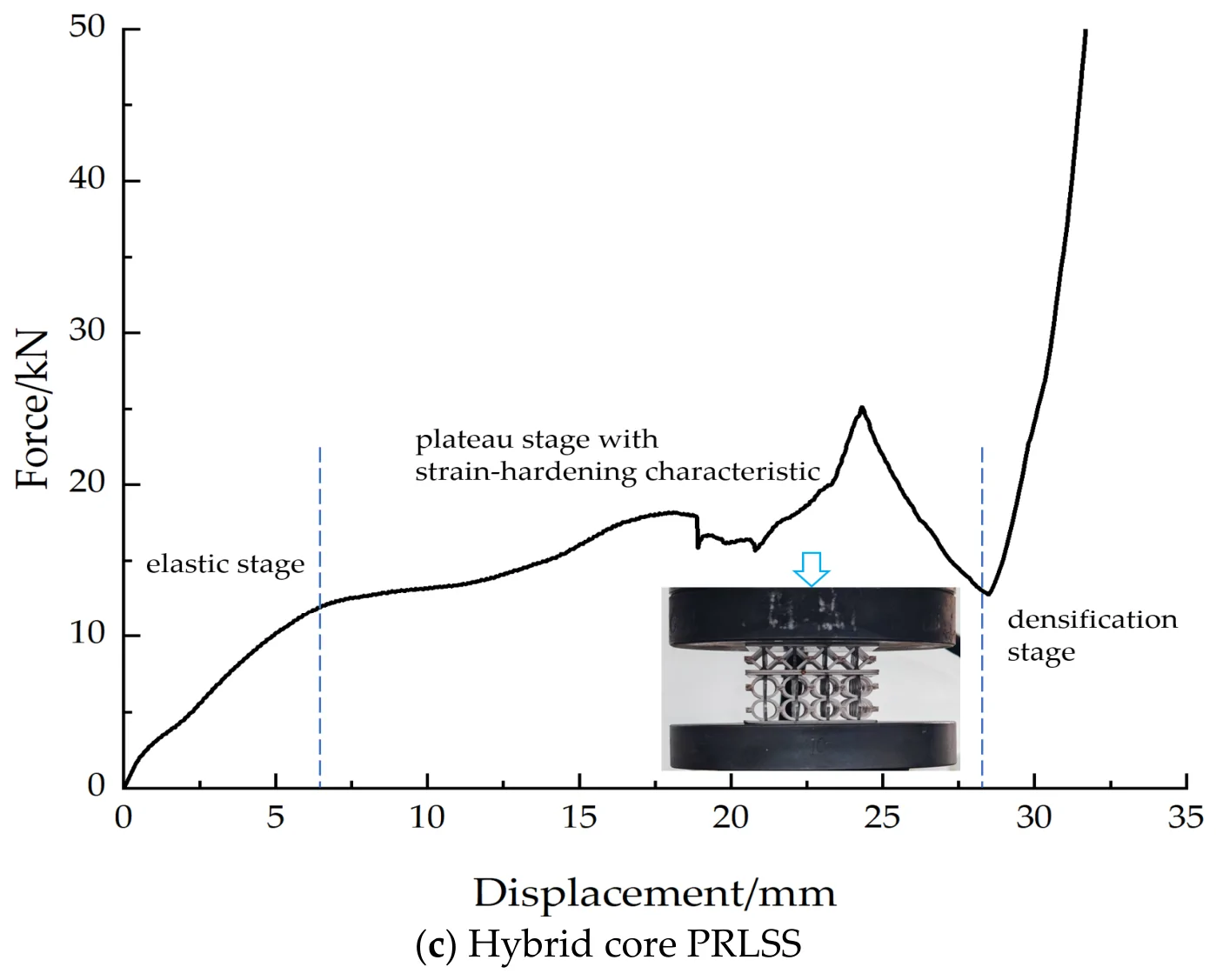
Load-displacement curves of quasi-static compressive tests.

**Figure 9:**
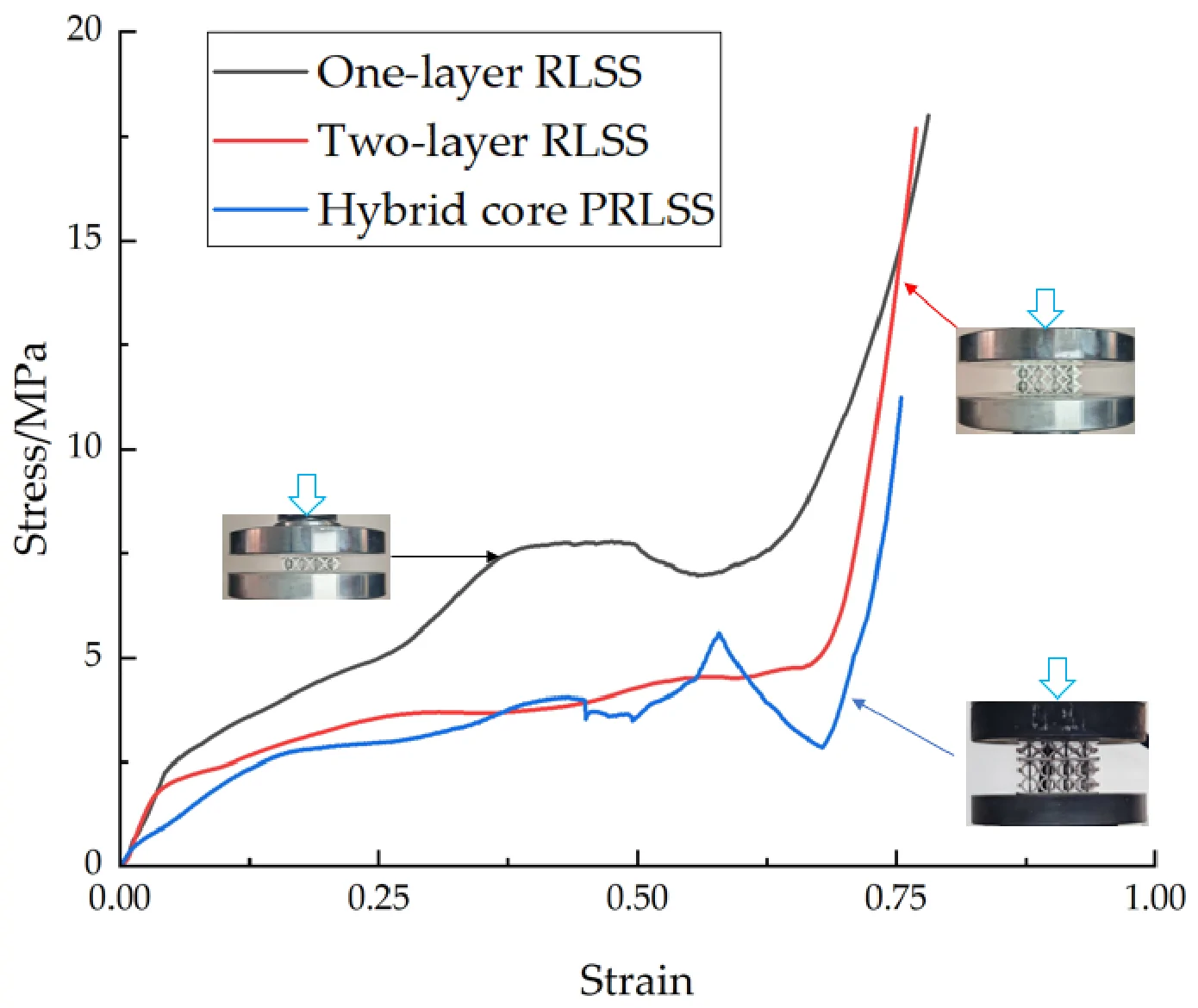
Quasi-static compressive stress–strain curves of RLSSs and hybrid core PRLSS.

The load-displacement curves and stress–strain curves obtained from the quasi-static compression tests are shown in Figure 8 and Figure 9. The load-displacement curves of the RLSSs can be divided into an elastic stage, a plateau stage with a strain-hardening characteristic and a densification stage. Furthermore, under the same strain condition, the stress of the one-layer RLSS is higher than that of the double-layer structure, because the unstable torsional deformation of rings at the contact points between the upper and lower core layers weakens the energy absorption capacity of the two-layer core. The hybrid core PRLSS exhibits an initial stress–strain response similar to the two-layer RLSS, followed by a later-stage response characterized by sharp fluctuations, which correspond to the buckling of the straight struts in the pyramidal layer.

### 2.4. SHPB Tests of the Ring Lattice Sandwich Structures

**Figure 10:**
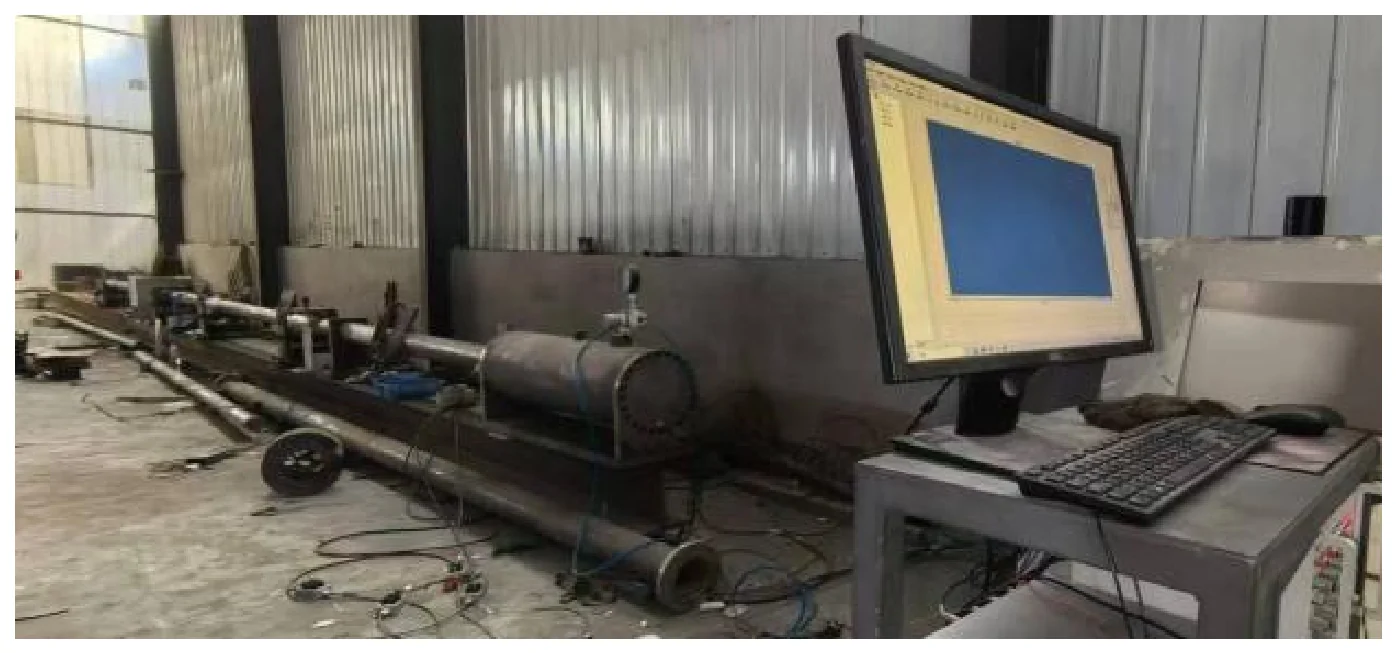
SHPB test apparatus.

The dynamic compressive mechanical properties of the ring lattice sandwich structures were tested using a ACHIMEDES Φ100 mm Split Hopkinson Pressure Bar (SHPB) apparatus (Archimedes Industry Technology Co., Ltd., Tianjin, China). The SHPB system consists of a pressure bar system, a loading/driving system, a data measurement system, and a data acquisition system. The pressure bar system is composed of a striker bar (projectile), an incident bar, a specimen, a transmission bar, and a damper, as shown in Figure 10. The pressure bar system is essentially an elastic system, in which each part of the bars is an elastic rod. Through loading and driving, the elastic bars transmit one-dimensional elastic stress waves to cause deformation and failure of the specimen. The dynamic compression process of the ring lattice sandwich structure is captured by a high-speed camera. Meanwhile, the data of elastic wave propagation are recorded by the measurement system on the bars, and the stress–strain curves of the specimen at a given strain rate are obtained after processing by the ACHIMEDES SHPB software (version 1.0). The striker bar (projectile), incident bar, and transmission bar are all made of spring steel. The dimensions are as follows: striker bar (projectile) Φ100 × 400 mm, incident bar Φ100 × 4830 mm, and transmission bar Φ100 × 2980 mm.

## 3. Energy Absorption Indicators

To objectively and quantitatively analyze the mechanical properties and energy absorption characteristics of the RLSSs, energy absorption indicators are used to analyze the data obtained from tests and numerical simulations. The energy absorption indicators selected in this paper are summarized as follows:

### 3.1. Initial Yield Stress $\sigma_{cr}$

$\sigma_{cr}$ marks the stress at the transition point from the elastic stage to the plastic stage on the compressive stress–strain curve of the lattice structure. The corresponding strain is called the yield strain $\varepsilon_{cr}$.

### 3.2. Densification Strain $\varepsilon_{cd}$

The strain at the transition point from the plateau stage to the densification stage on the compressive stress–strain curve of the lattice structure is called the densification strain $\varepsilon_{cd}$. The stress corresponding to the densification strain $\varepsilon_{cd}$ is denoted as $\sigma_{cd}$. There are many methods for determining the densification strain, such as the function of relative density method, the tangent intersection technique, and energy absorption efficiency diagram.

According to the function of relative density, the densification strain $\varepsilon_{cd}$ can be expressed as [22]:

(3) $$\varepsilon_{cd}=1-\alpha \frac{\rho_{0}}{\rho_{s}}$$

where $\rho_{0}$ is the original density of the lattice structure, $\rho_{s}$ is the density of the solid phase, and α is a coefficient that needs to be determined experimentally.

**Figure 11:**
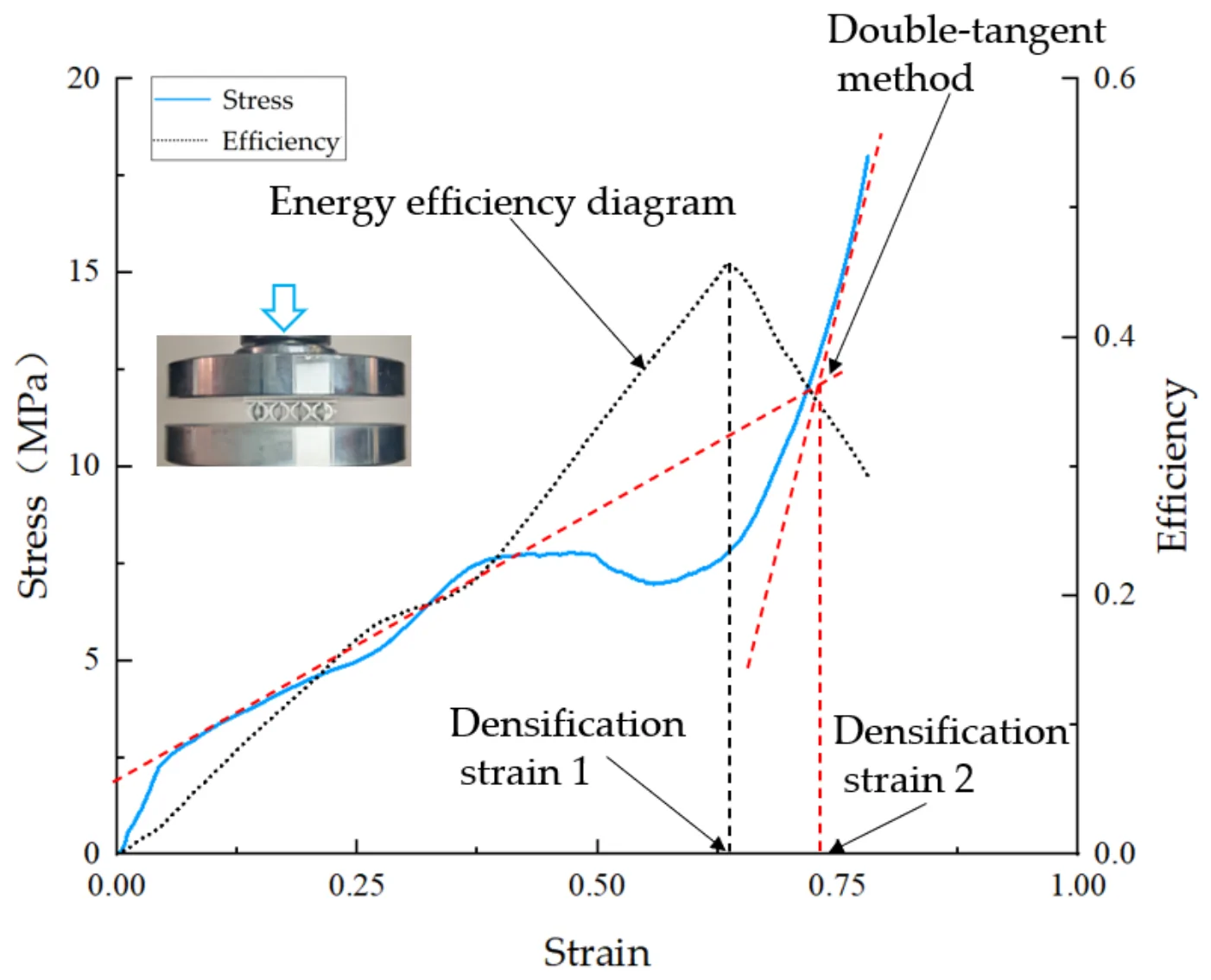
Comparison of densification strains obtained by the double-tangent method and the energy efficiency diagram.

According to the tangent intersection technique, the densification strain is defined as the intersection of the tangents to the stress plateau regime and densification regime [23]. Based on the energy absorption efficiency diagram, the strain corresponding to the position of the maximum energy absorption efficiency on the energy absorption efficiency vs. strain curve is taken as the densification strain $\varepsilon_{cd}$ [24]. To illustrate the difference between these two definitions, the one-layer RLSS is examined in Figure 11. The densification strains obtained by the tangent intersection method and the energy efficiency diagram are 0.73 and 0.63, respectively, with a relative difference of 15.8%. The corresponding absorbed energies are 261.98 J and 206.39 J, showing a discrepancy of 26.9%.

The relative-density-based method requires empirical calibration of coefficients, and the tangent intersection method involves ambiguity in tangent placement. The energy absorption efficiency diagram, however, is simpler to implement and provides densification strains that are consistent with the stress–strain response, supporting its validity.

### 3.3. Energy Absorption Efficiency (E)

Energy absorption efficiency refers to the ratio of the energy absorbed by the lattice structure at a given strain ε to the energy absorbed by an ideal energy absorber producing the same peak stress [22]. It is given by:

(4) $$E=\frac{\int_{0}^{\varepsilon }\sigma \left(\varepsilon \right)d\varepsilon }{\sigma_{p}*1}$$

where $\sigma_{p}$ refers to the peak stress of the lattice structure up to the given strain ε.

### 3.4. Plateau Stress $\sigma_{pl}$

The plateau stress $\sigma_{pl}$ is the average stress over the plateau stage from the yield strain $\varepsilon_{cr}$ to the densification strain $\varepsilon_{cd}$ [24], given by:

(5) $$\sigma_{pl}=\frac{\int_{\varepsilon_{cr}}^{\varepsilon_{cd}}\sigma \left(\varepsilon \right)d\varepsilon }{\varepsilon_{cd}-\varepsilon_{cr}}$$

where $\sigma \left(\varepsilon \right)$ refers to the stress of the lattice structure up to the given strain ε.

### 3.5. Plateau Stress Fluctuation (PSF)

PSF refers to the degree of stress fluctuation over the plateau stage from the yield strain $\varepsilon_{cr}$ to the densification strain $\varepsilon_{cd}$ [25], given by:

(6) $$\mathrm{PSF}=\frac{\int_{\varepsilon_{cr}}^{\varepsilon_{cd}}\left|\sigma \left(\varepsilon \right)-\sigma_{pl}\right|d\varepsilon }{\int_{\varepsilon_{cr}}^{\varepsilon_{cd}}\sigma \left(\varepsilon \right)d\varepsilon }$$

### 3.6. Energy Absorption (EA)

EA refers to the energy absorbed during the crushing process of the lattice structure [25], given by:

(7) $$\mathrm{EA}=\int_{0}^{H}F(x)dx=V\int_{0}^{\varepsilon_{cd}}\sigma (\varepsilon )d\varepsilon$$

where *F*(*x*) is the compressive load corresponding to a given displacement *x*, *H* is the displacement corresponding to the densification strain $\varepsilon_{cd}$, and *V* is the volume of the lattice structure.

### 3.7. Specific Energy Absorption (SEA)

SEA refers to the energy absorbed per unit mass of the lattice structure [26], given by:

(8) $$\mathrm{SEA}=\frac{\int_{0}^{H}F(x)dx}{m}=\frac{\int_{0}^{\varepsilon_{cd}}\sigma (\varepsilon )d\varepsilon }{\rho }$$

where *m* is the mass of the lattice structure and *ρ* is the density of the lattice structure.

## 4. Numerical Simulation

### 4.1. Finite Element Model

**Figure 12:**
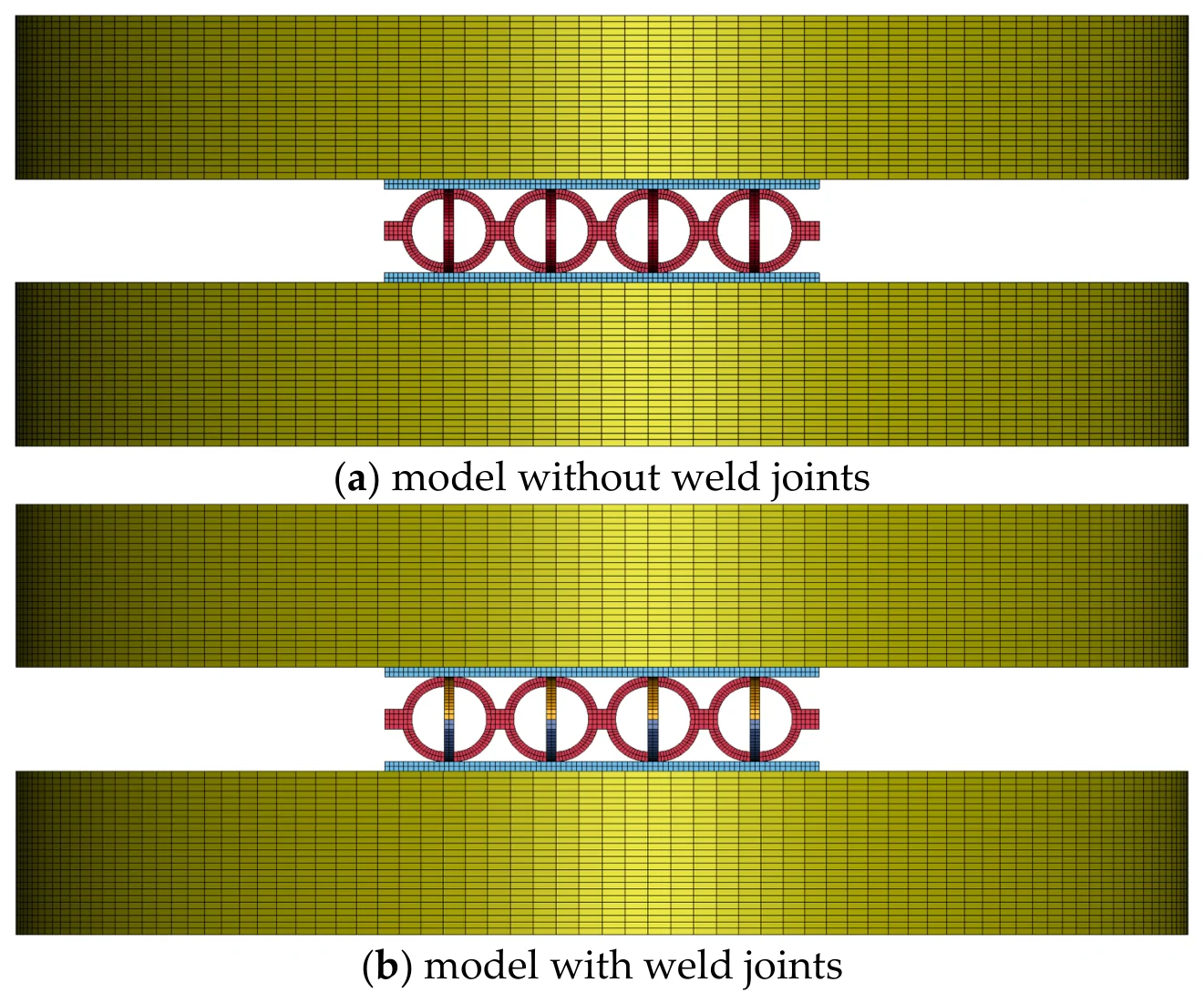
Quasi-static compressive models of RLSSs with and without weld joints.

Due to the complex process, long preparation time, and high cost of the ring lattice sandwich structure, it is difficult to cover all working conditions through experiments. Therefore, numerical simulation methods were used to further study the mechanical properties and energy absorption characteristics of the ring lattice structure. The “mass scaling method” was employed to numerically simulate the quasi-static compression process of the structure using ANSYS/LS-DYNA software (version 17.0) [27]. To analyze the influence of the weld joints on the local stiffness, stress distribution, and deformation patterns of the specimen, a numerical model for quasi-static compression of the one-layer RLSS with and without weld joints was established, as shown in Figure 12. The core, panels, and platens were all modeled using SOLID164 elements. The welding at the contact areas between the core and panels was treated using shared nodes. Based on the quasi-static tensile stress–strain curve characteristics of the furnace-accompanied base material, the *MAT_PLASTIC_KINETIC material model was used for the core and panels, and the key parameters were equal to those in Table 1. To eliminate inertial effects, the C and P values of the material model were set to 0. The *MAT_RIGID material model was used for the platens. *Automatic_Surface_to_Surface contact was used between the platen and the panel, and between the panel and the core. *Automatic_Single_Surface contact was used for the core self-contact. For the RLSS numerical model with joints, the contact between the interlocking strips is defined using *Automatic_Surface_to_Surface_Tiebreak, and the normal failure stress of the BNi-2 brazed seam is 300 MPa [28].

**Figure 13:**
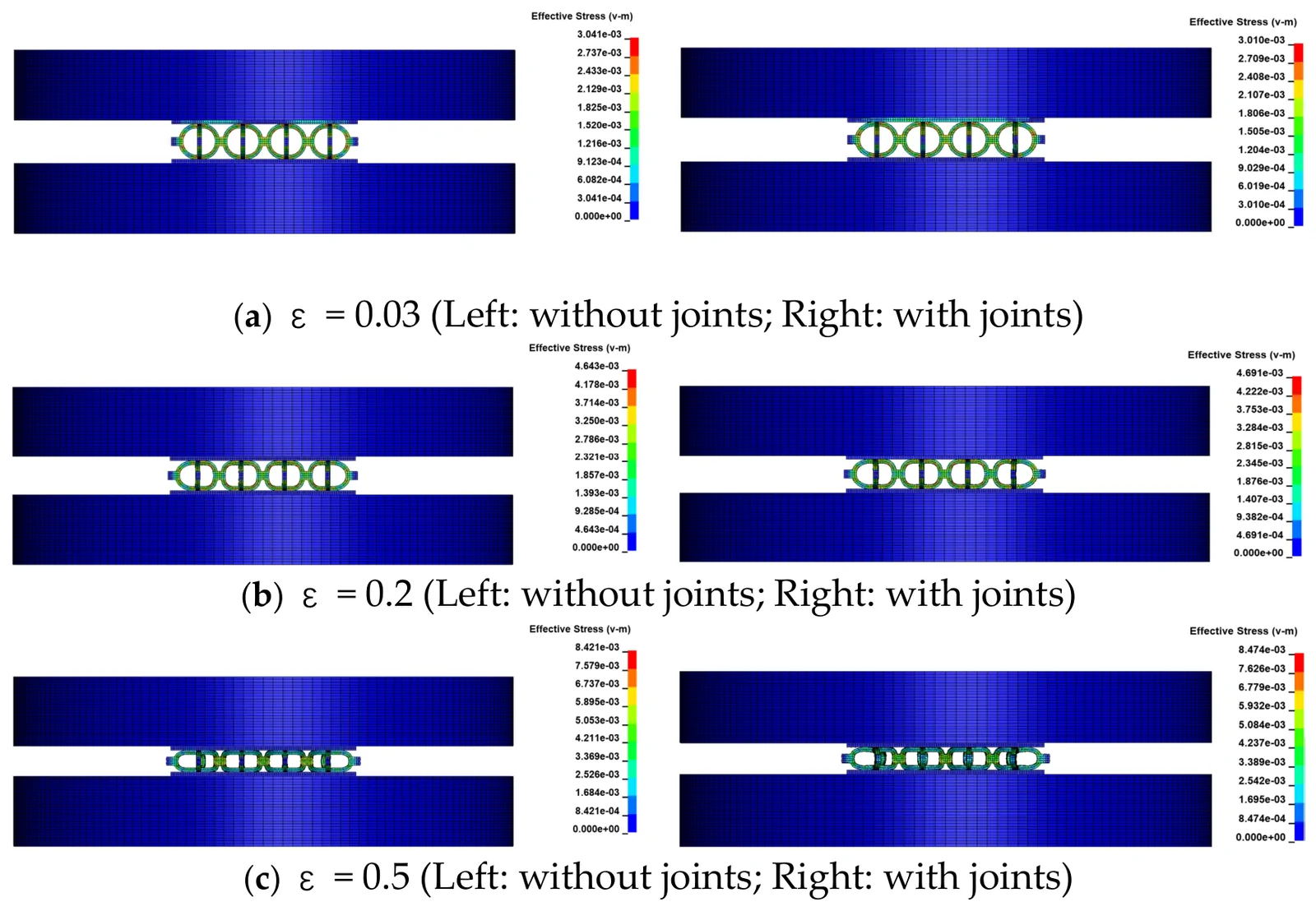
Quasi-static stress contour of RLSSs with and without joints.

**Figure 14:**
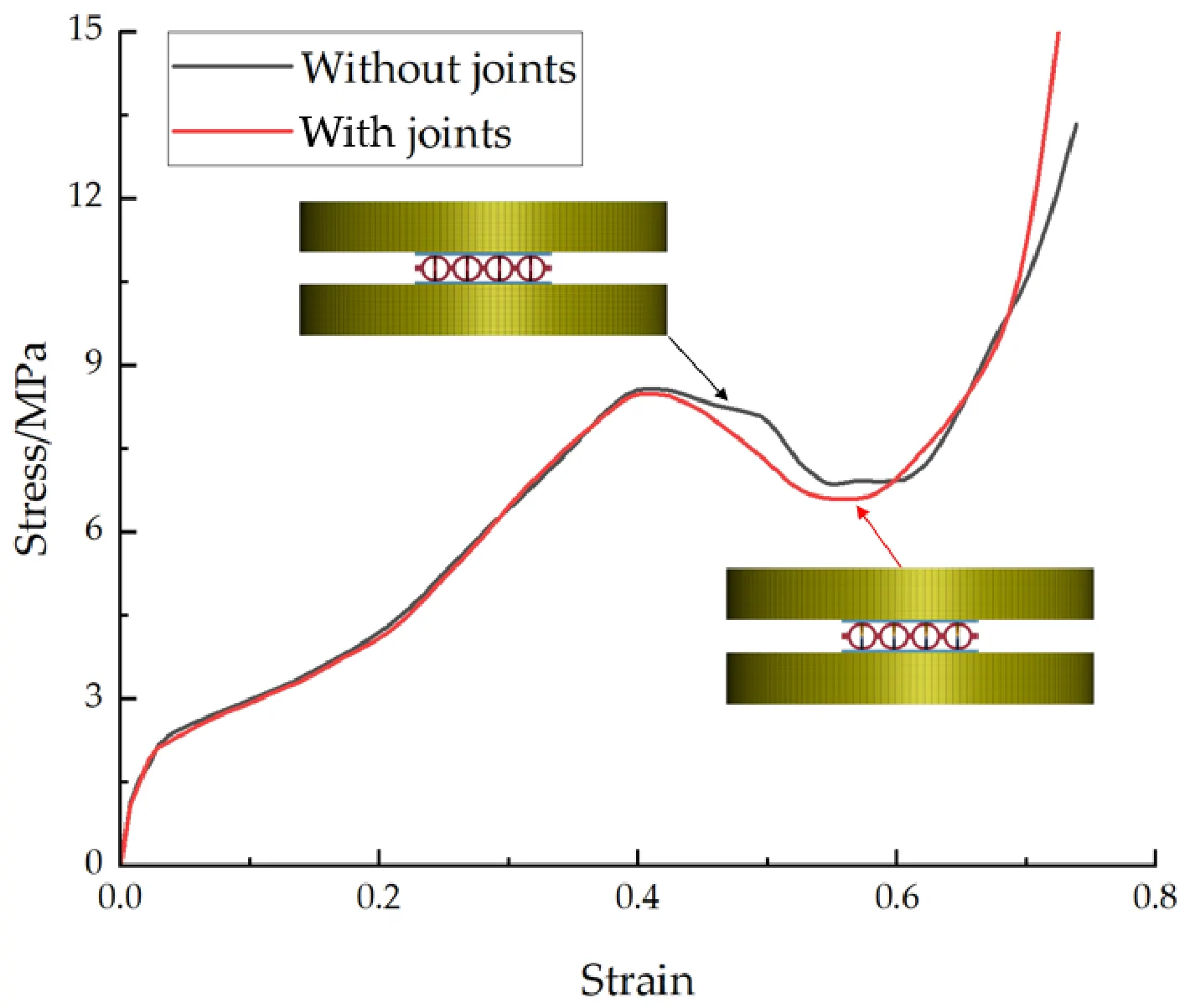
Quasi-static compressive stress–strain curves of RLSSs with and without joints.

The stress contour plots and stress–strain curves from the quasi-static compressive numerical simulations of the one-layer RLSS with and without weld joints are shown in Figure 13 and Figure 14, respectively. As can be seen from Figure 13, under the same strain rate, the structural deformations are essentially consistent, the stress distributions are similar, and the weld joints have little effect on the deformation mode and stress distribution of the RLSS. Figure 14 shows that the quasi-static compressive stress–strain curves of the RLSSs with and without weld joints are in good agreement. Combining the metallographic micrographs of the weld joints and the quasi-static compressive numerical simulation results of the one-layer RLSS with and without weld joints, the vacuum-brazed RLSS structure exhibits good overall integrity. The influence of the weld joints on the structure can be neglected, and the ring core layer can be simplified as an integral whole.

### 4.2. Mesh Convergence Analysis

**Figure 15:**
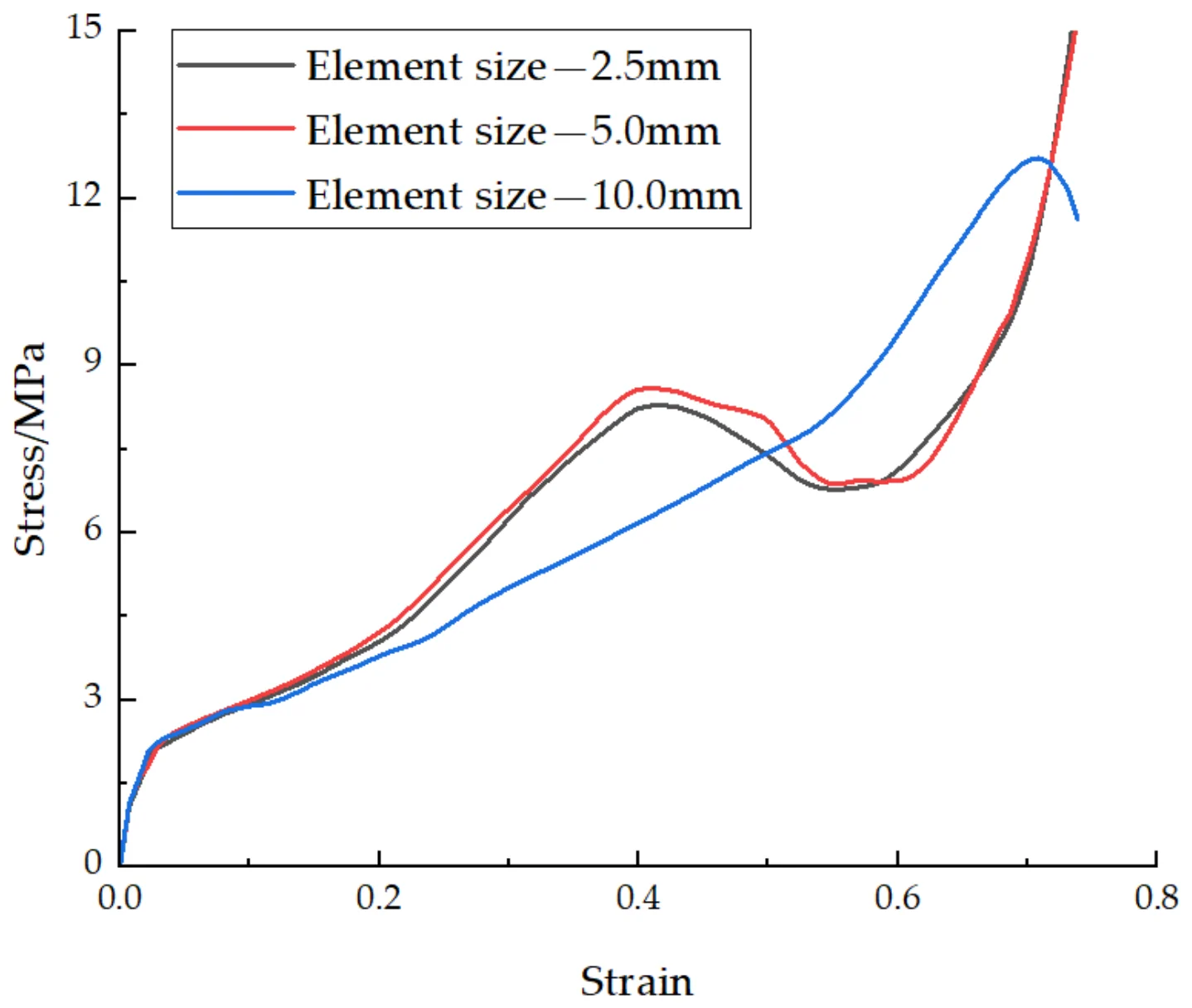
Quasi-static stress–strain curves of RLSSs with different element sizes.

Mesh size significantly influences calculation time and the simulation result. Generally, a finer mesh size produces a more precise result although it increases calculation time [29]. To achieve a balance between computational accuracy and computational time, a mesh convergence analysis was conducted on the quasi-static compressive numerical model of the one-layer RLSS. Mesh convergence tests were performed by halving the element sizes, with element sizes set to 10.0 mm, 5.0 mm, and 2.5 mm, respectively. The stress–strain curves and energy absorption parameters obtained from the numerical simulations are presented in Figure 15 and Table 2. It can be observed from Figure 15 that the stress–strain curves for element sizes of 5.0 mm and 2.5 mm are in good agreement, whereas they differ considerably from the curve for the 10.0 mm element size. Further analysis from Table 2 reveals that when the element size is halved from 10.0 mm to 5.0 mm, the differences in plateau yield stress and energy absorption are 11.4% and 24.5%, respectively, indicating that convergence has not yet been achieved. When the element size is further halved from 5.0 mm to 2.5 mm, the differences are 2.5% and 4.3%, respectively, which can be considered as convergence. Therefore, in this study, the element size is set to 5.0 mm to strike an appropriate balance between computational accuracy and computational time.

### 4.3. Model Validation

**Figure 16:**
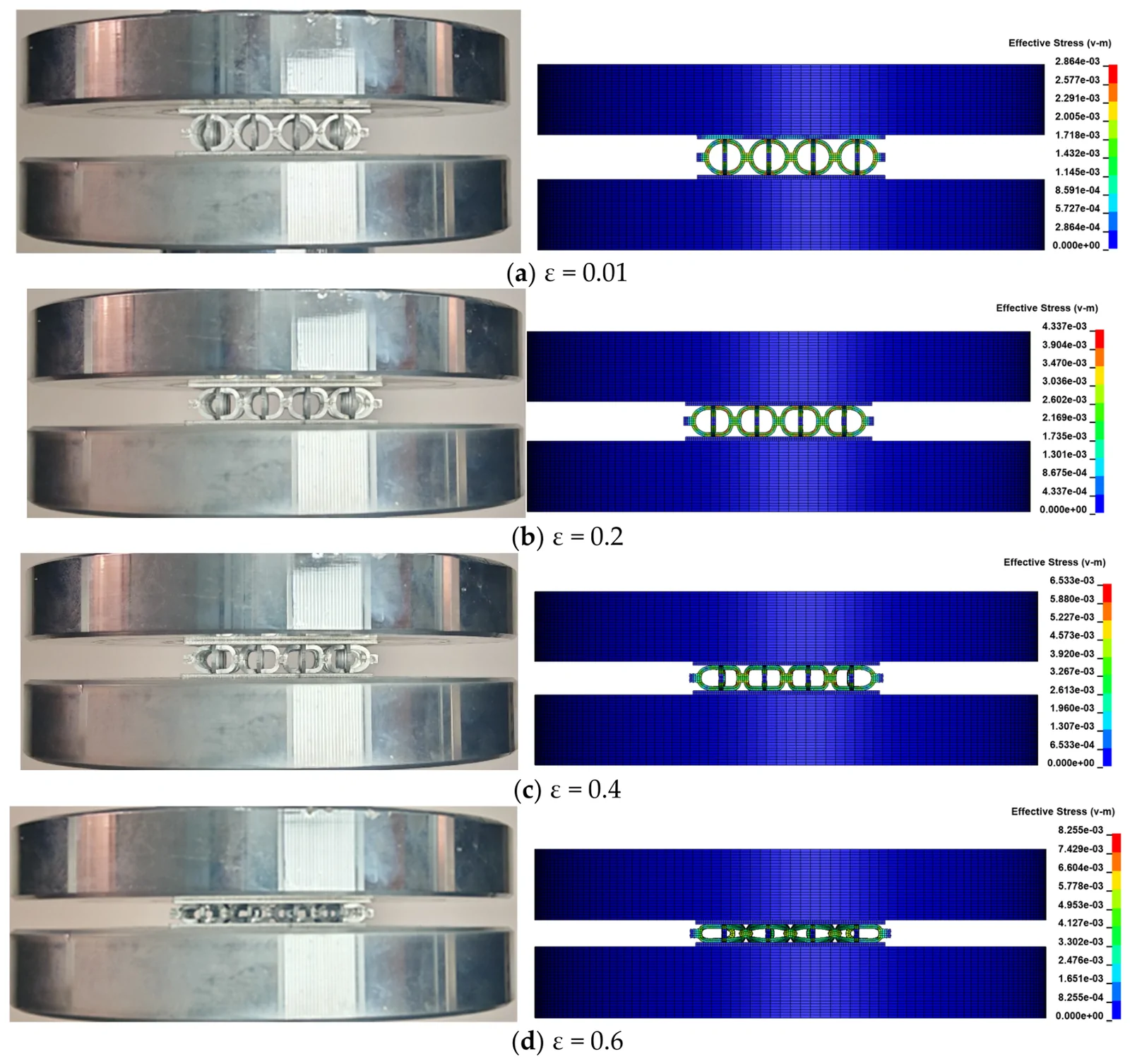
Comparison of quasi-static compression process between test and numerical simulation for RLSS (4 × 4 × 1).

**Figure 17:**
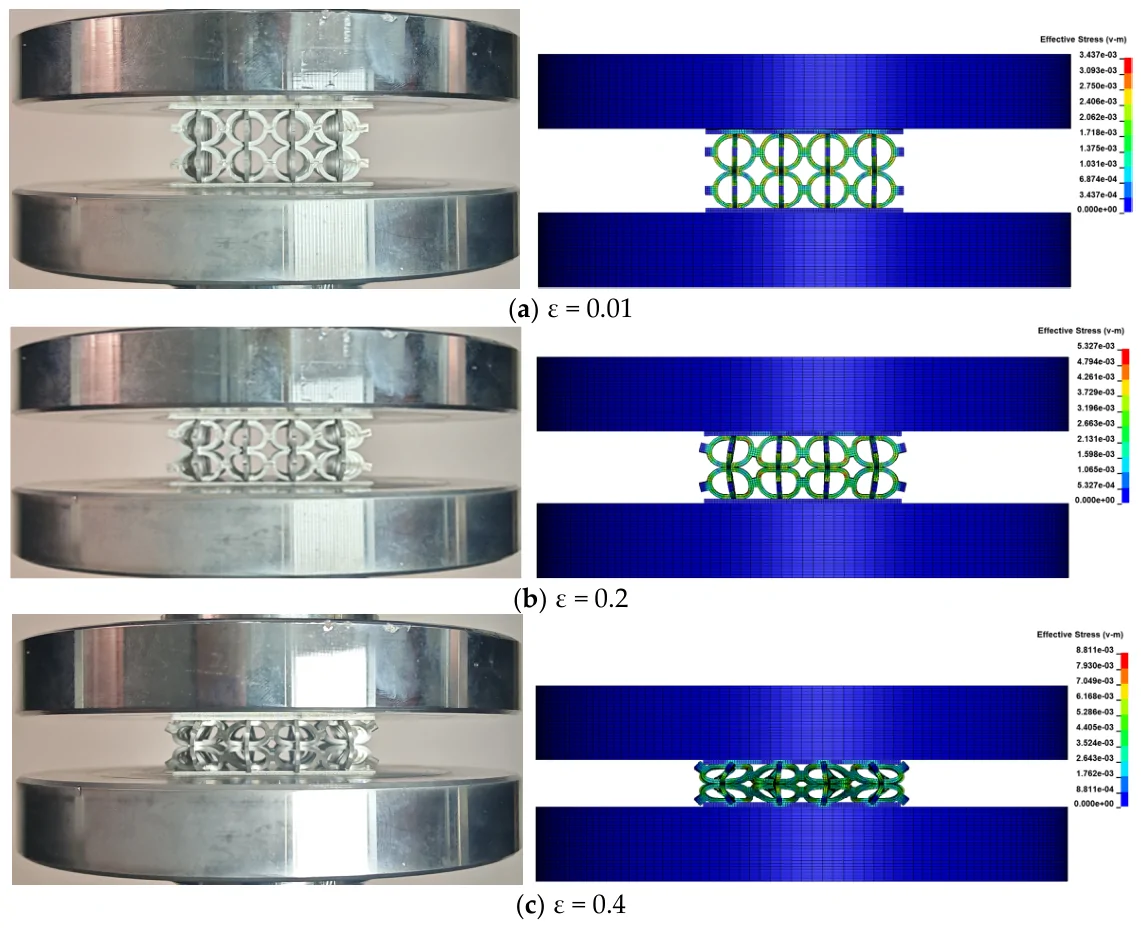

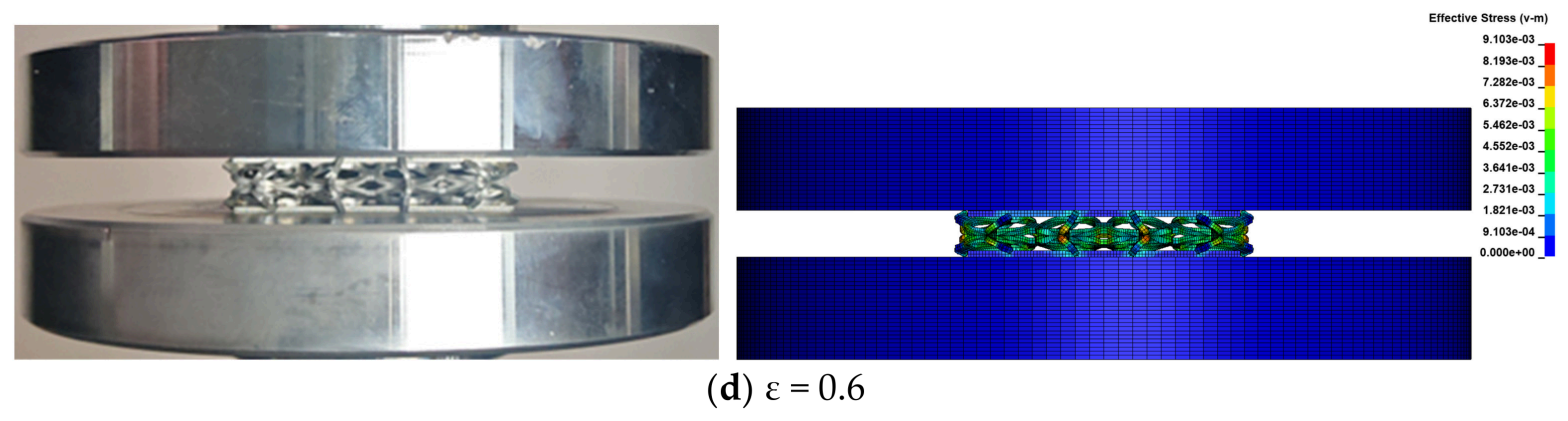
Comparison of quasi-static compression process between test and numerical simulation for RLSS (4 × 4 × 2).

**Figure 18:**
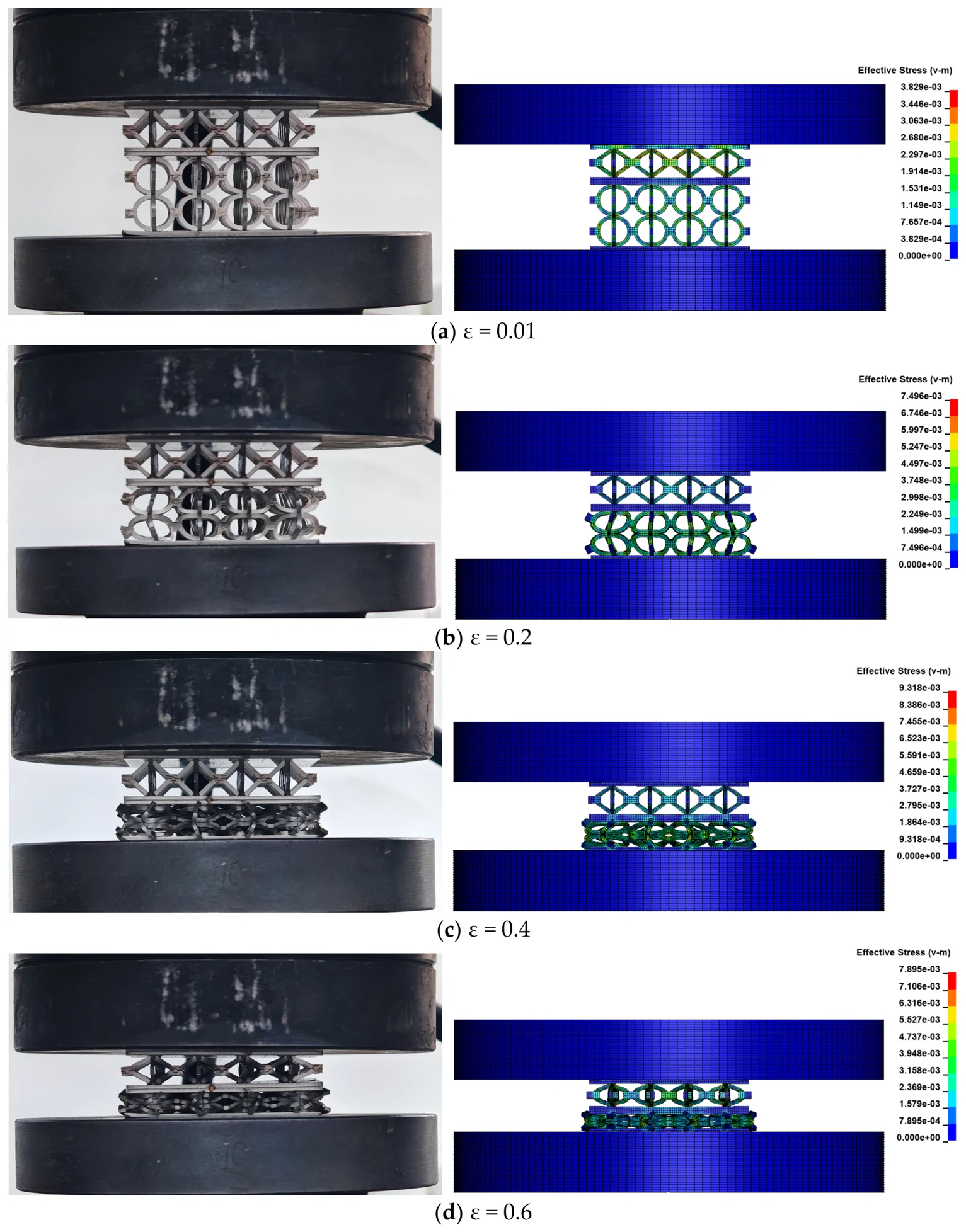
Comparison of quasi-static compression process between test and numerical simulation for the hybrid core PRLSS.

**Figure 19:**
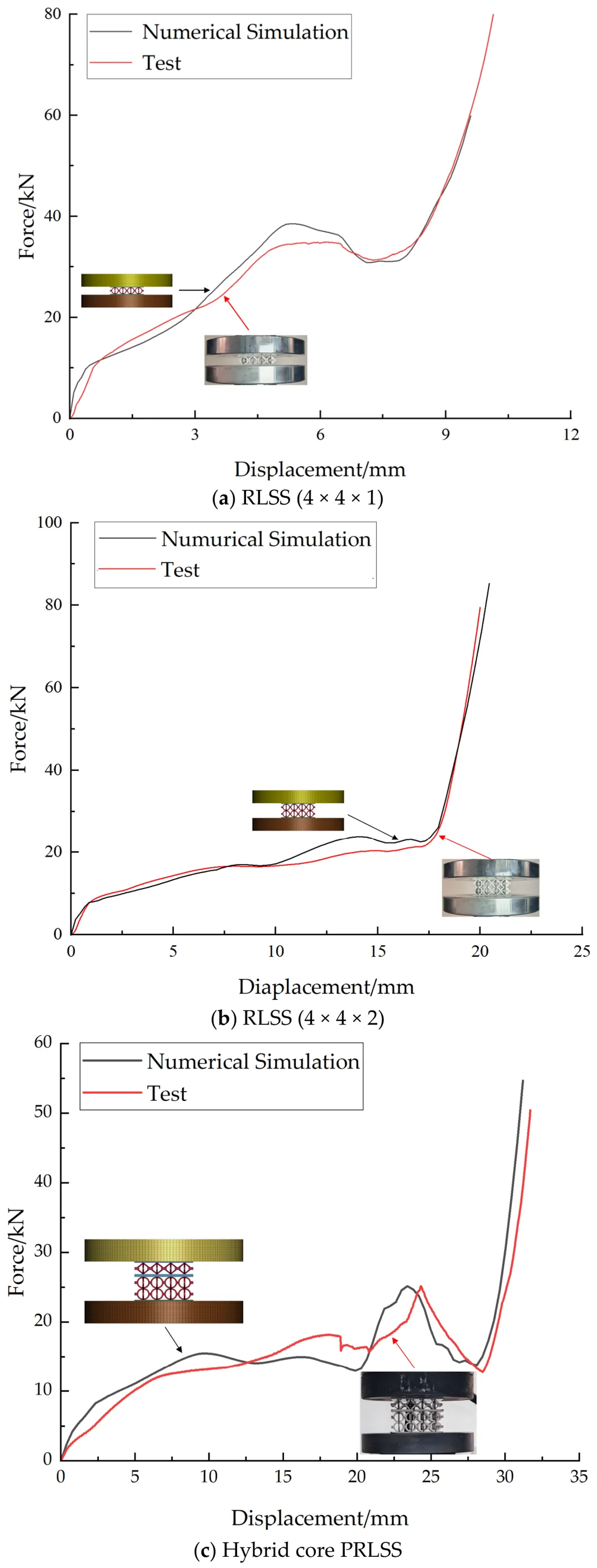
Quasi-static compressive load-displacement curves from tests and simulations for one-layer RLSS, two-layer RLSS, and hybrid core PRLSS.

**Figure 20:**
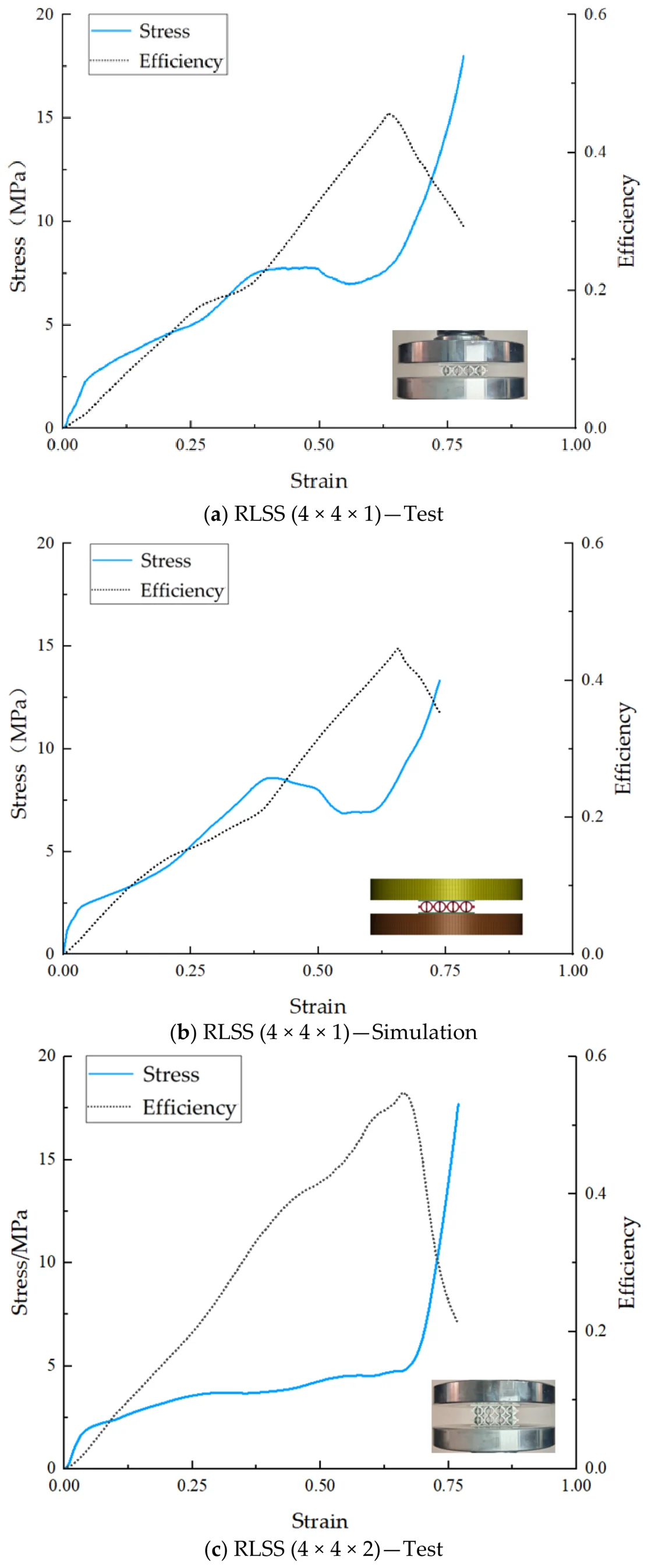

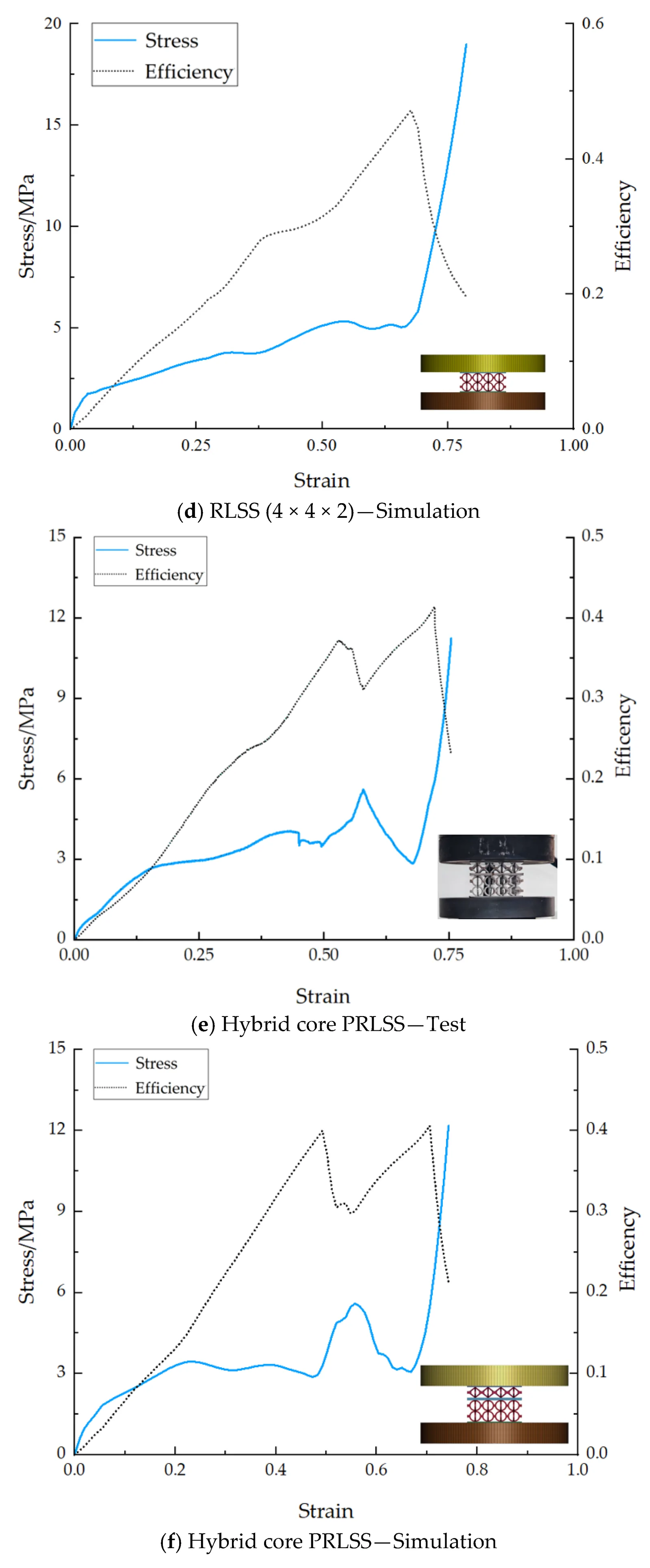
Energy absorption efficiency diagrams from tests and simulations for one-layer and two-layer RLSSs under quasi-static compression.

The quasi-static compression process, load-displacement curves, and energy absorption efficiency diagrams obtained from tests and numerical simulations are shown in Figure 16, Figure 17, Figure 18, Figure 19 and Figure 20, and the energy absorption indicators are listed in Table 3. It can be seen from Figure 16, Figure 17 and Figure 18 that the numerical simulation accurately reproduces the quasi-static compression process of one-layer RLSS, two-layer RLSS, and hybrid core PRLSS. The one-layer structure crushes vertically, while the two-layer structure exhibits torsional deformation at the contact points between the upper and lower core layers. Analyzing Figure 19, the yield loads obtained from the test and simulation for the one-layer structure are 10.0 kN and 9.8 kN, respectively, with a relative error of 2.0%; for the two-layer structure, they are 8.3 kN and 7.8 kN, with a relative error of 6.2%. Further analysis from Figure 20 and Table 3 shows that the indicators (yield strain, densification strain, yield stress, plateau stress, plateau stress fluctuation, energy absorption, and specific energy absorption) obtained from tests and simulations for one-layer RLSS, two-layer RLSS and hybrid core PRLSS are in good agreement. For the hybrid-core PRLSS, the relative errors in densification strain, plateau stress, energy absorption efficiency, and energy absorption are only 1.4%, 0.29%, 0%, and 1.3%, respectively. The excellent agreement across all these key parameters provides strong evidence for the effectiveness of the numerical model.

The experimental observations (Figure 16, Figure 17 and Figure 18) and numerical energy analysis (Table 4) together provide a comprehensive picture of the deformation and energy dissipation mechanisms. The one-layer RLSS exhibits a relatively uniform stress distribution, with peak stresses concentrated at nodes and curved-strut mid-spans, consistent with its vertical crushing mode. In contrast, both the two-layer RLSS and hybrid-core PRLSS show peak stresses mainly at the severely twisted mid-span regions of the curved struts, corresponding to the torsional deformation at layer interfaces. Quantitatively, LS-DYNA results (Table 4) indicate that core plastic deformation is overwhelmingly dominant in energy absorption, contributing more than 85% of the total dissipated energy, while friction accounts for approximately 5%, with other mechanisms contributing only marginally. This confirms that core crushing is the primary energy absorption mechanism in the ring-based lattice sandwich structures.

### 4.4. Numerical Simulation Working Conditions

**Figure 21:**
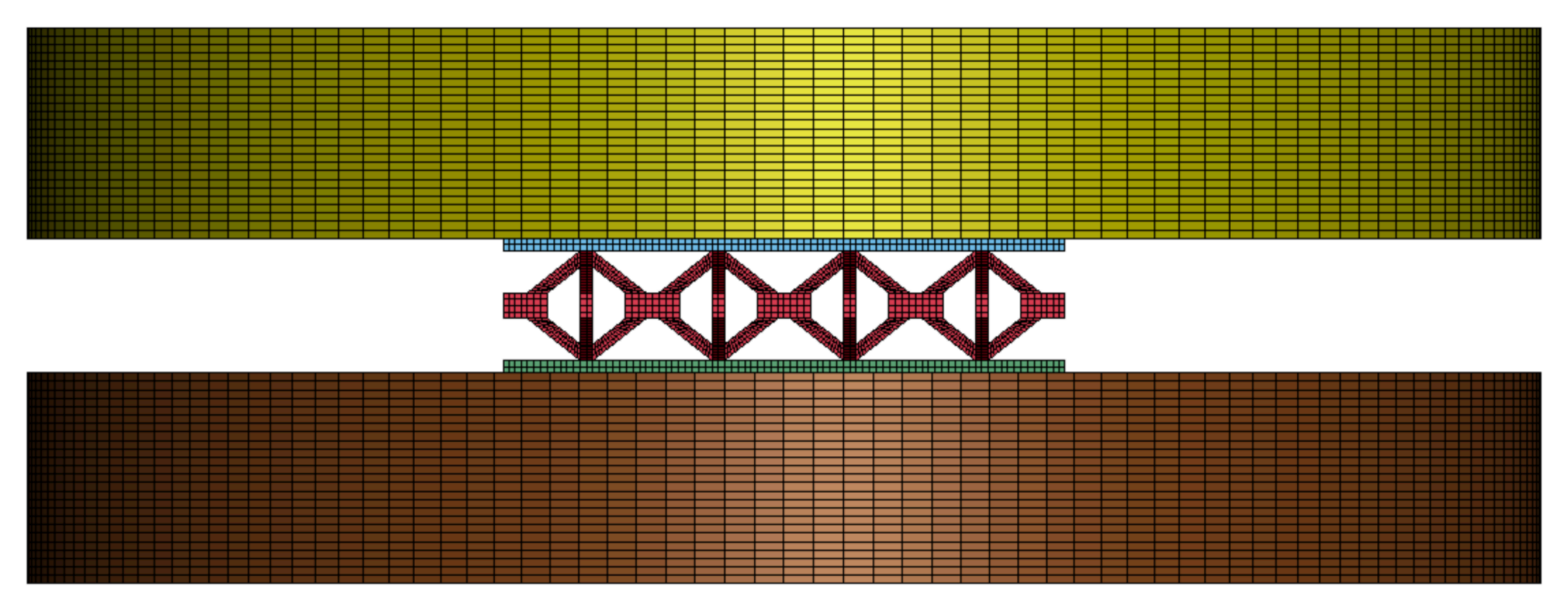
Numerical model for quasi-static compression of PLSS (4 × 4 × 1).

**Figure 22:**
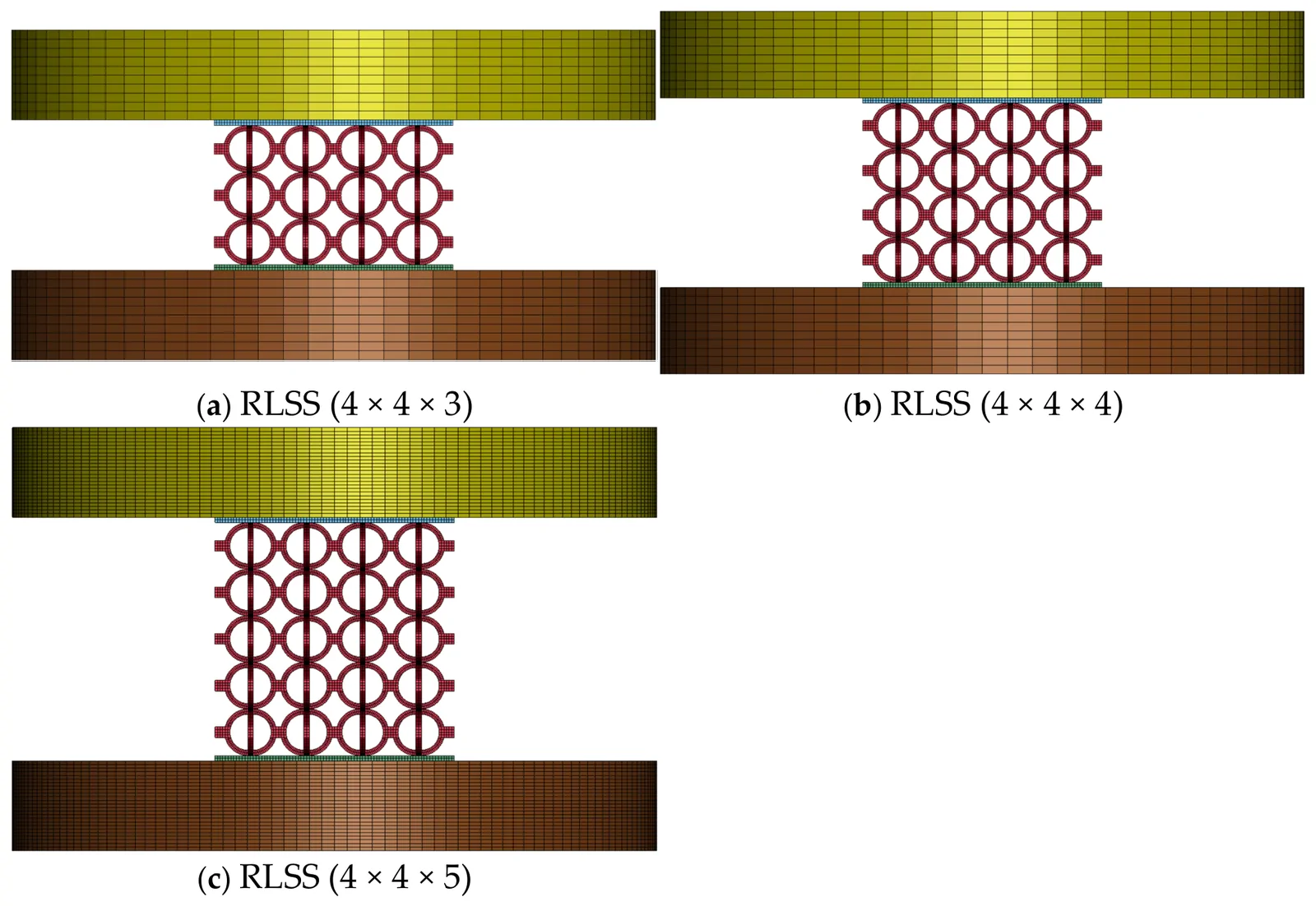
Numerical models for quasi-static compression of RLSSs with different layers.

**Figure 23:**
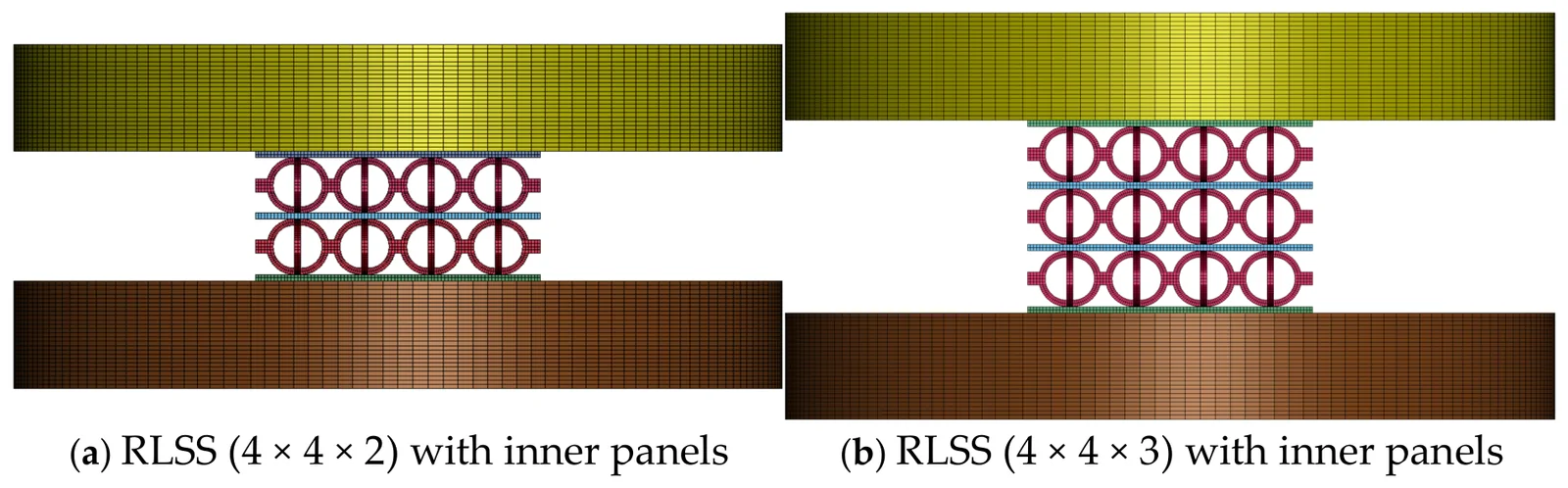
Numerical models for quasi-static compression of RLSSs with inner panels.

**Figure 24:**
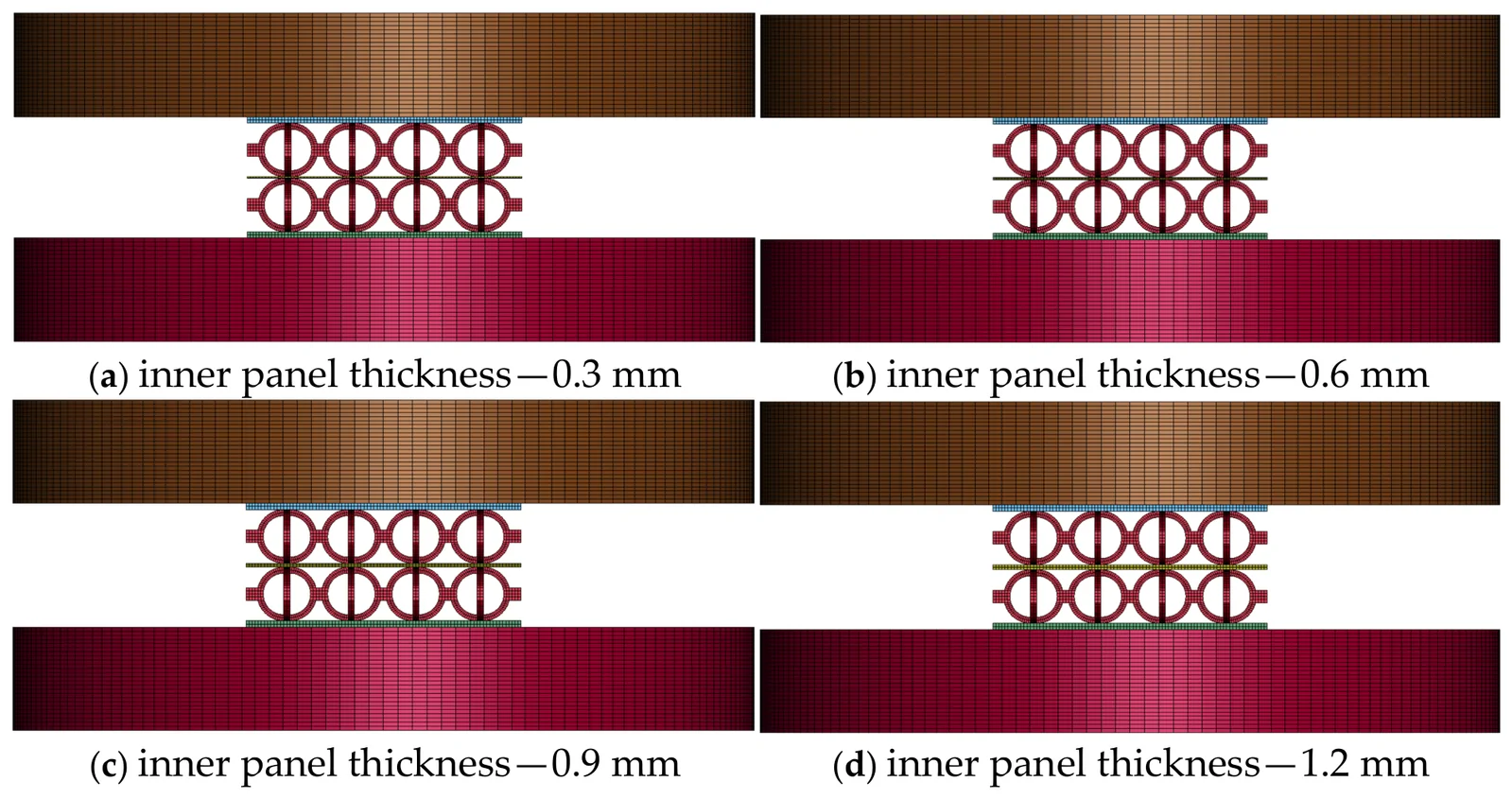
Numerical models for quasi-static compression of RLSS (4 × 4 × 2) with inner panels of different thicknesses.

**Figure 25:**
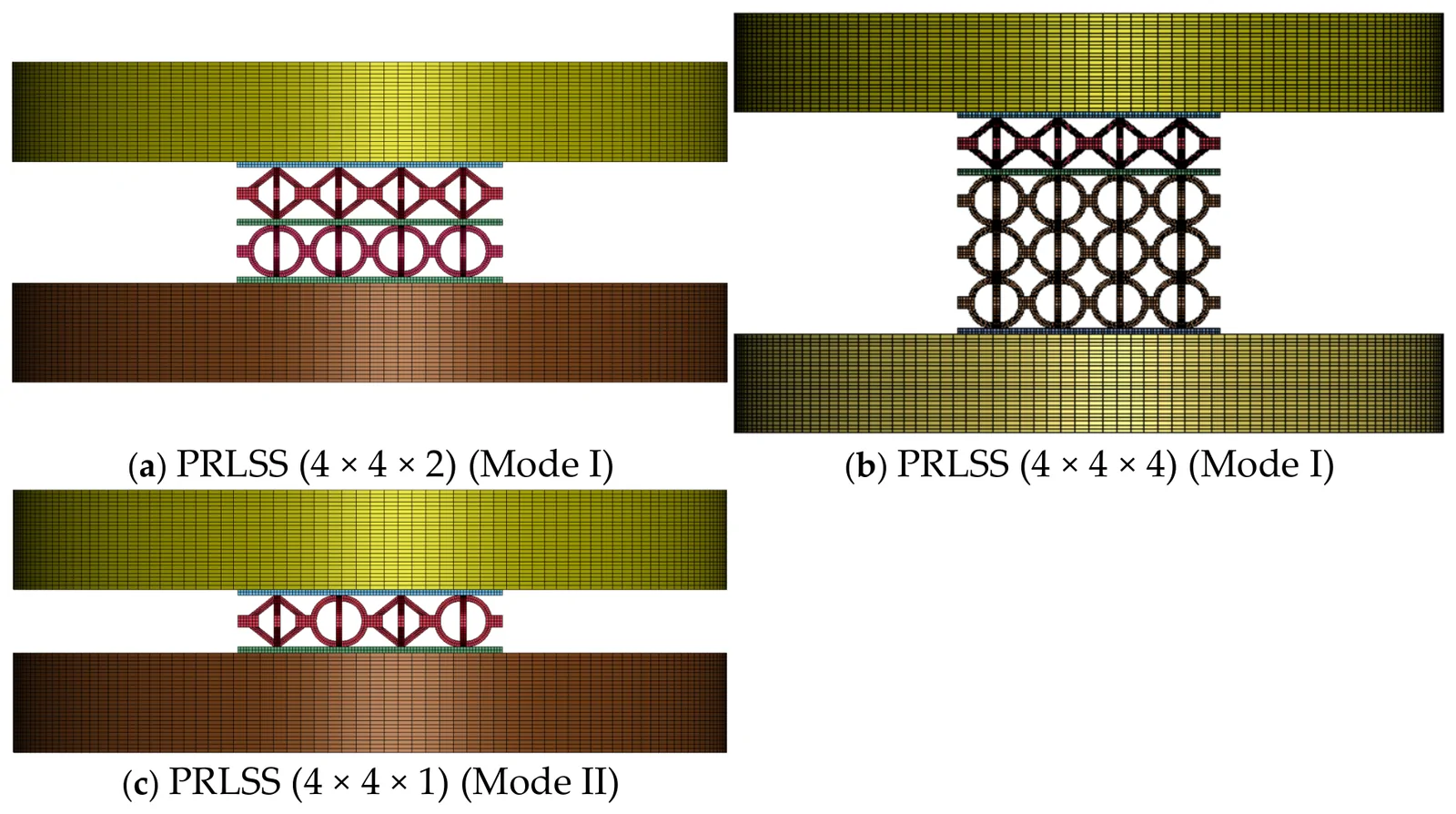
Numerical models for quasi-static compression of hybrid ring and pyramidal lattice sandwich structures.

To further explore the mechanical properties and energy dissipation mechanisms of the RLSSs, numerical simulation studies were carried out under the following four working conditions based on the validated numerical model: (a) Quasi-static compression numerical simulation of PLSS (4 × 4 × 1) (Figure 21); (b) Quasi-static compression numerical simulations of RLSS with different core layers (Figure 22); (c) Quasi-static compression numerical simulations of RLSS (4 × 4 × 2) with inner panels and RLSS (4 × 4 × 3) with inner panels (Figure 23); (d) Quasi-static compression numerical simulations of RLSS (4 × 4 × 2) with inner panels of different thicknesses (Figure 24); (e) Quasi-static compression numerical simulations of hybrid ring and pyramid lattice sandwich structures (Figure 25).

## 5. Theoretical Analysis

### 5.1. Dimensionless Relative Density

**Figure 26:**
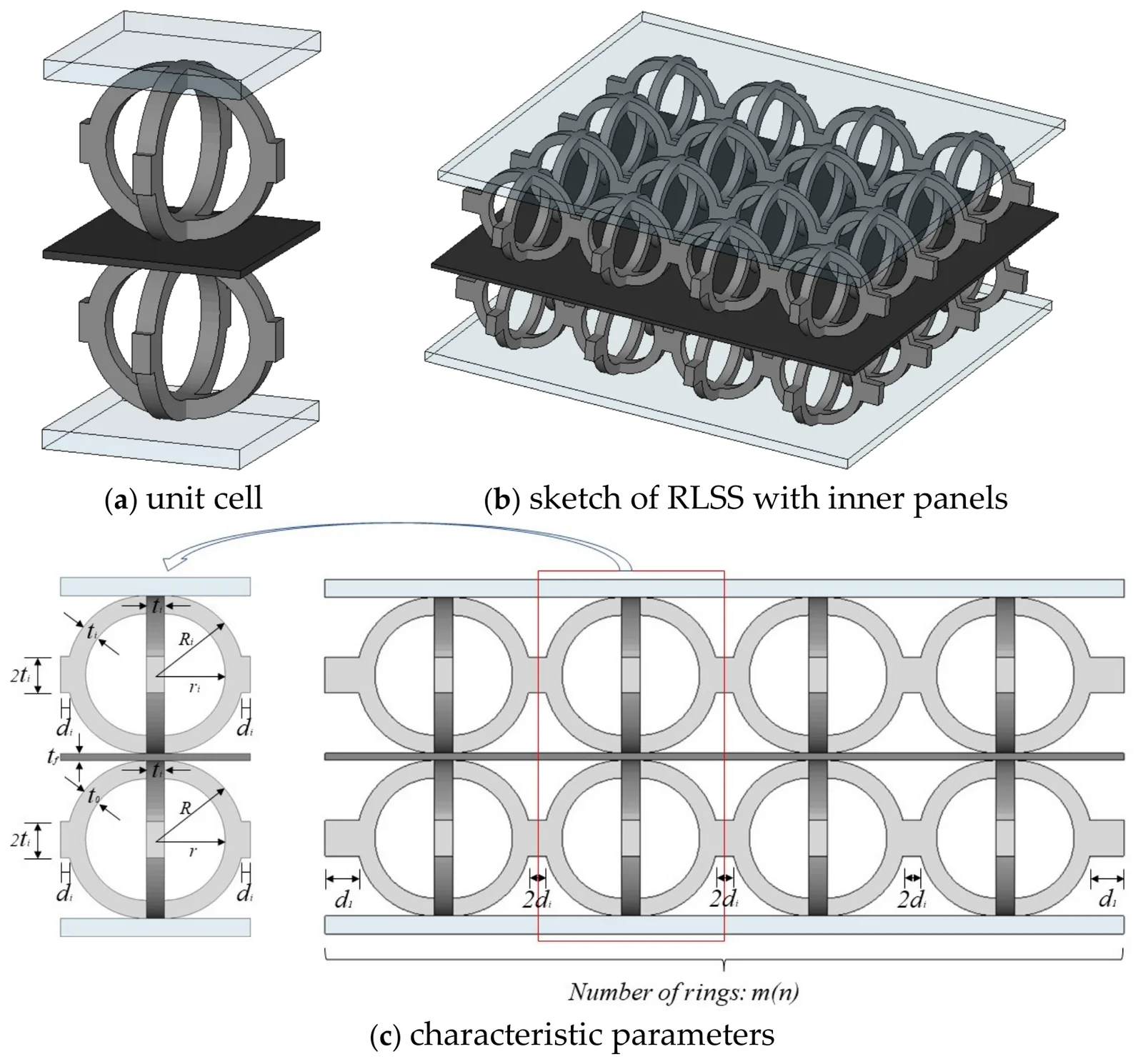
Geometric model and characteristic parameters of RLSS with inner panels.

Taking a typical RLSS (m × n × k) with inner panels as an example, m, n, and k represent the number of rings in the meridional, latitudinal, and height directions, respectively, as shown in Figure 26. The outer diameter of the i-th layer of the circular ring is *R_i_*, the inner diameter is *r_i_*, the thickness of the interlocking strip and the length of the contact position between the interlocking strip and the panel are both *t_i_*, the length of the joint of the middle circular ring is 2*d_i_*, the length of the joint of the edge circular ring is *d*_1_, the height of the joint of the circular ring is 2*t_i_*, and the thickness of the middle panel is *t_f_*. For simplified calculation, assuming the length of the joint of the edge ring is *d_i_*, the relative density of the one-layer core is equal to the relative density of a single cell of the ring in that layer, which can be expressed as:

(9) $${\bar{\rho }}_{i}=\frac{2[2\pi \frac{\left(R_{i}+R_{i}-t_{i}\right)}{2}t_{i}+4t_{i}d_{i}]t_{i}+2t_{i}^{2}}{2(R_{i}+d_{i})\cdot 2(R_{i}+d_{i})\cdot 2R_{i}}$$

Simplification yields:

(10) $${\bar{\rho }}_{i}=\frac{[\pi (2R_{i}-t_{i})+4d_{i}+1]t_{i}^{2}}{4R_{i}{\left(R_{i}+d_{i}\right)}^{2}}$$

The dimensionless relative density of the core layer of RLSS (m × n × k) with inner panels can be expressed as:

(11) $$\bar{\rho }=\frac{\sum_{1}^{k}{\bar{\rho }}_{i}h_{i}+(k-1)t_{f}}{\sum_{1}^{k}h_{i}+(k-1)t_{f}}$$

where *h_i_* represents the height of the circular ring of the *i*-th core layer.

Taking the RLSS (4 × 4 × 2) with a 1.5 mm thick inner panel as an example, the outer diameter is 6.5 mm. The thickness of the interlocking strip and the length of the contact position between the interlocking strip and the panel are both 1.5 mm. The length of the intermediate circular joint is 1.5 mm, the height at the circular joint is 3.0 mm, and the thickness of the inner panel is 1.5 mm. The theoretically calculated relative density is 11.7%, while the relative density obtained from the numerical model is 12.6%. The relative error between theoretical and numerical simulation is 7.1%, indicating a good degree of agreement between the two, which verifies the effectiveness of the dimensionless relative density model.

### 5.2. Initial Yield Stress

Due to the complexity of compressive deformation of the multi-layer RLSSs, which involves torsional deformation during the crushing process, only the crushing behavior of the one-layer ring lattice structure will be analyzed here. Depending on the constraints, the crushing modes of the circular ring lattice structure can be categorized into edge ring crushing and middle ring crushing.

#### 5.2.1. Initial Yield Load of Edge Rings [30,31]

**Figure 27:**
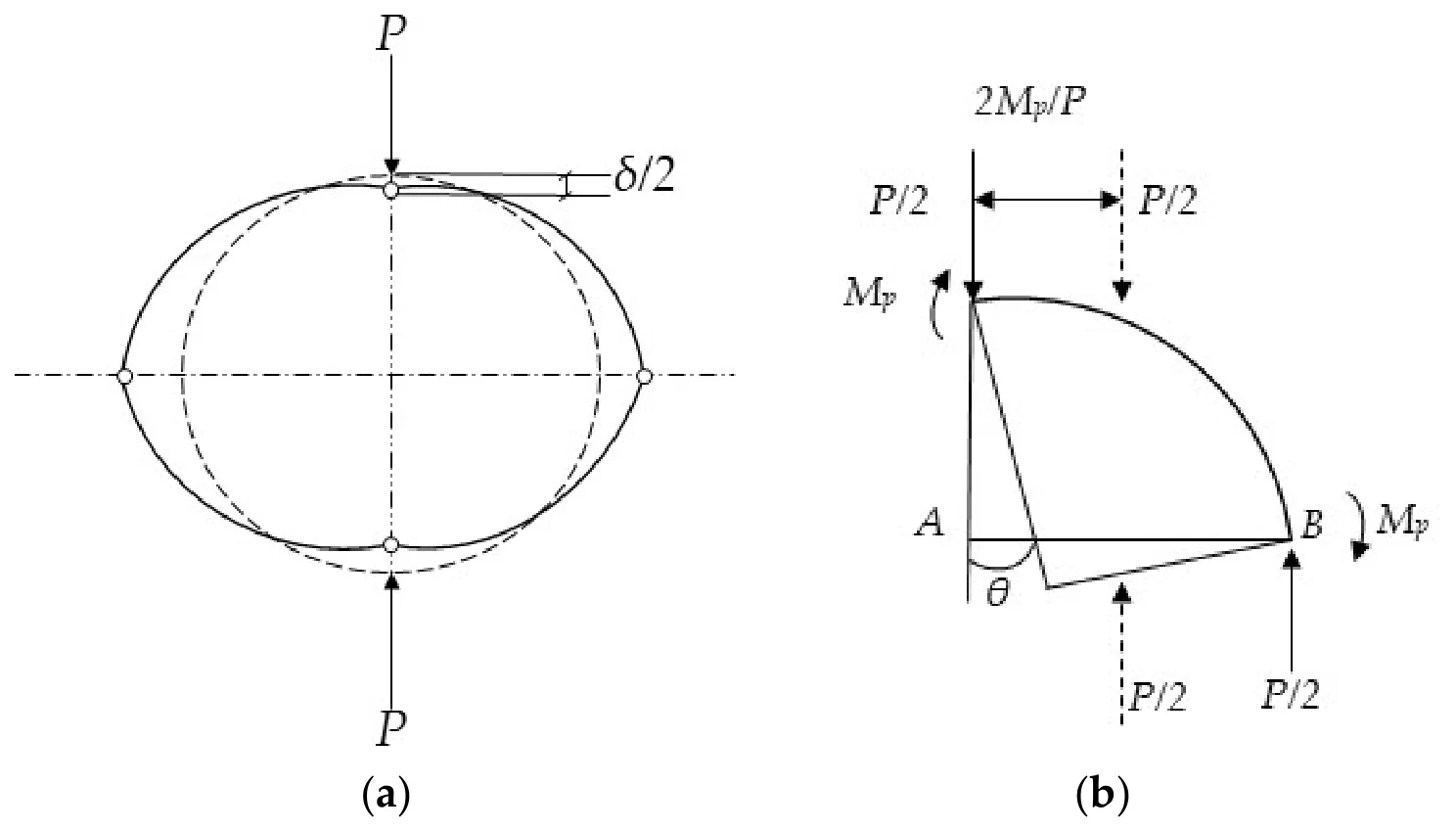
Collapse mechanism of a compressed circular ring under a pair of inwardly concentrated forces. (**a**) Initial collapse mechanism of four plastic hinges. (**b**) Force diagram acting on a quarter of the ring.

For the edge rings, their deformation mode can be regarded as a ring compressed by two plates, and the initial collapse load is the same as that of a compressed ring under the action of a concentrated force. For a rigid-plastic ring with a radius of *R*_0_ and a thickness and width of *t* in its cross-section, under the action of a pair of concentrated forces in opposite directions, four plastic hinges are required to form a collapsed structure, as shown in Figure 27.

For the derivation process, please refer to Appendix A.1.

The initial yield load *P*_01_ can obtained as follows:

(12) $$P_{01}=\frac{4M_{p}}{R_{0}}=\frac{\sigma_{0}t^{3}}{R-t/2}$$

#### 5.2.2. Initial Yield Load of Middle Rings [32]

**Figure 28:**
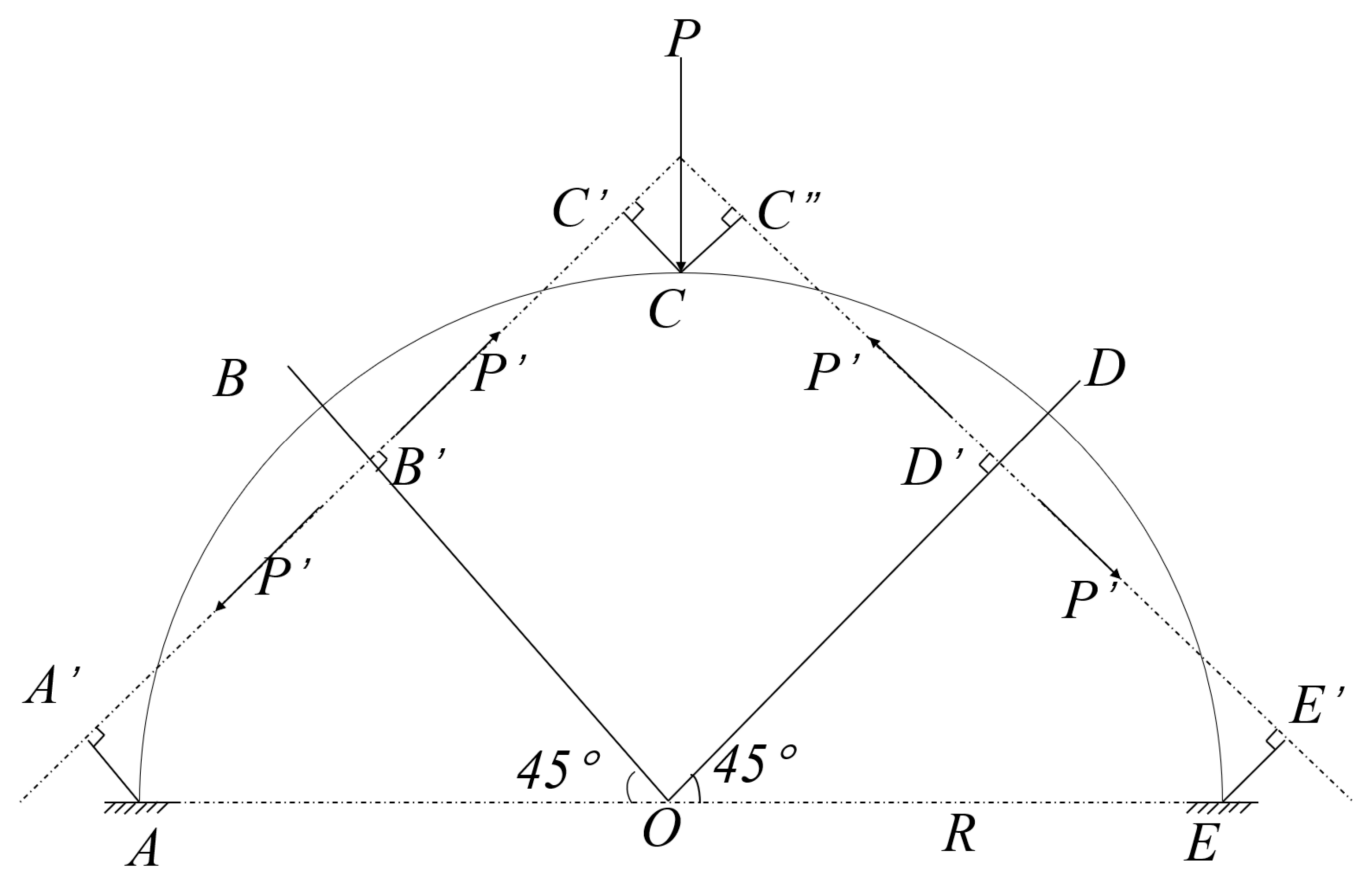
Initial collapse mechanism of a built-in semi-circular arch under inward concentrated load.

For the middle rings, their initial collapse load is approximately similar to that of a built-in semi-circular arch under the action of a concentrated force. Consider a built-in semicircular arch with a radius of *R*_0_, a section thickness and width both of *t*, and a plastic bending moment of *M_p_*. Neglecting the influence of axial force, under the inward concentrated load *P*, five plastic hinges are required to form a collapsed structure, as shown in Figure 28.

For the derivation process, please refer to Appendix A.1.

The initial yield load *P*_02_ can be obtained:

(13) $$P_{02}=\frac{4M_{p}}{R_{0}}(1+\sqrt{2})=(1+\sqrt{2})\frac{\sigma_{0}t^{3}}{R-t/2}$$

Comparing Formula (13) with Formula (12), it can be seen that under the same conditions, the initial yield load of the middle ring is 2.44 times that of the edge ring.

#### 5.2.3. Initial Yield Stress of RLSS

Taking RLSS (m × n × 1) as an example, its initial yield load *P*_0_ can be expressed as:

(14) $$P_{0}=N_{1}P_{01}+N_{2}P_{02}=\left[2(1+\sqrt{2})mn-\sqrt{2}(m+n)\right]\frac{\sigma_{0}t^{3}}{R-t/2}$$

The initial yield stress *σ_ini_* of RLSS (m × n × 1) can be obtained:

(15) $$\sigma_{ini}=\frac{P_{0}}{L_{m}L_{n}}=\frac{\left[2(1+\sqrt{2})mn-\sqrt{2}(m+n)\right]\sigma_{0}t^{3}}{4mn(R-t/2){\left(R+d\right)}^{2}}$$

For RLSS (m × n × 1) with large m and n, Equation (15) can be simplified as:

(16) $$\sigma_{ini}=\frac{1+\sqrt{2}}{2}\frac{\sigma_{0}t^{3}}{(R-t/2){\left(R+d\right)}^{2}}$$

As can be seen from Equation (16), the key parameters determining the initial yield stress of RLSS (m × n × 1) are the yield strength $\sigma_{0}$ of the ring material, the outer diameter *R* of the ring, the thickness *t* of the ring, and the connection length *d* between rings. For the same material, the initial yield stress of the ring lattice structure increases with the increase of the ring thickness *t*, and decreases with the increase of the outer diameter *R* of the ring and the connection length *d* between rings.

## 6. Results and Discussion

In this paper, different types of RLSSs were fabricated using the cutting-and-assembling method. Based on quasi-static tests and numerical simulations, the effects of different factors on the compressive mechanical properties and energy absorption characteristics of the RLSSs were analyzed.

### 6.1. Number of Ring Layers

**Figure 29:**
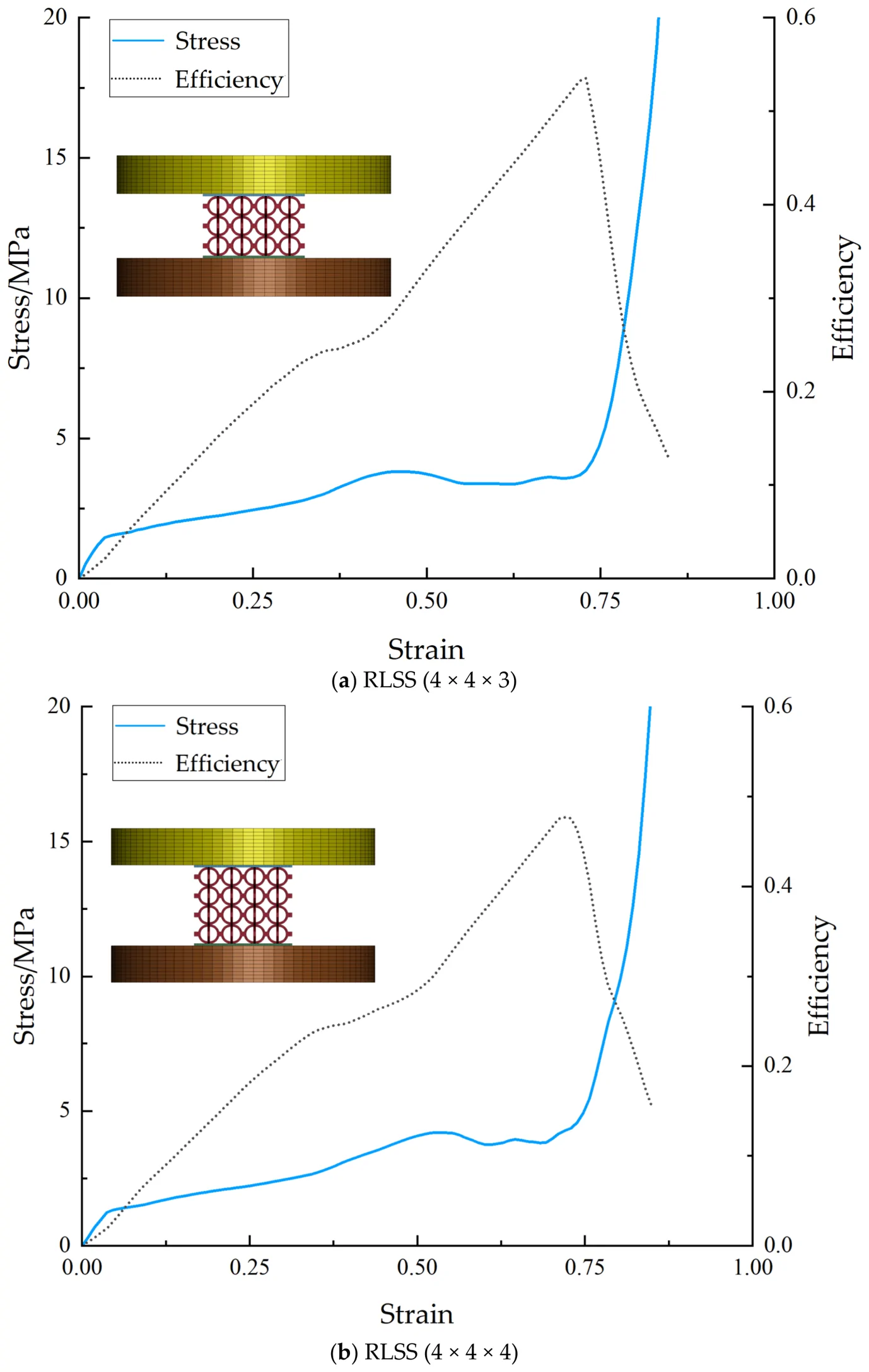

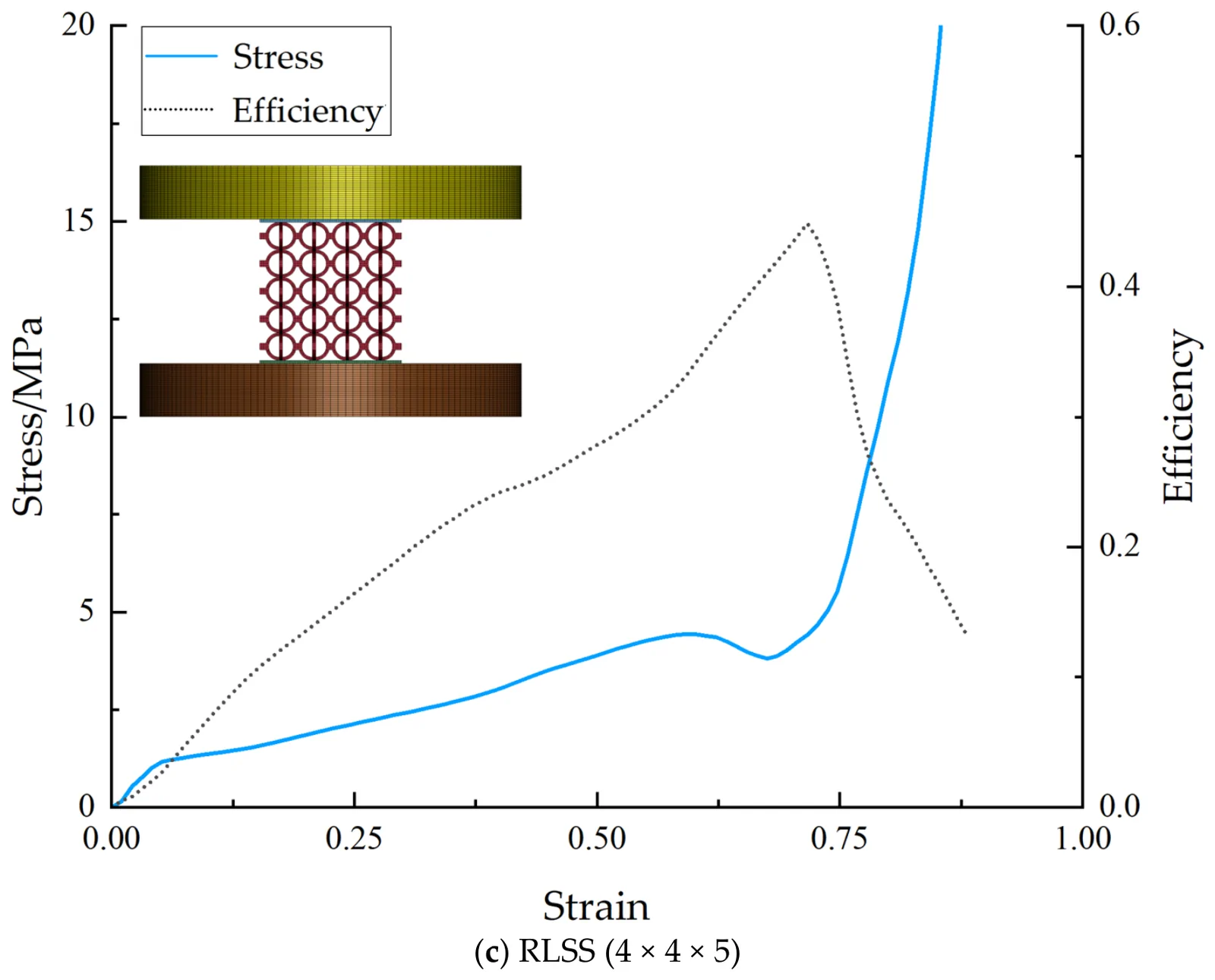
Energy absorption efficiency diagrams for three-layer, four-layer and five-layer RLSSs under quasi-static compression obtained from numerical simulation.

The energy absorption efficiency diagrams and indicators obtained from numerical simulations for RLSSs with different numbers of core layers are shown in Figure 20 and Figure 29, and Table 5. From Figure 20 and Figure 29, unlike the one-layer structure which shows a significant stress decrease before densification, the two-layer to five-layer structures exhibit essentially the same trend in their stress–strain curves during quasi-static compression: entering a strengthening stage after yielding, with stress increasing steadily with strain until a sharp increase upon reaching densification. The yield strains of the one-layer to five-layer RLSSs are 0.03, 0.03, 0.04, 0.04, and 0.05, respectively, showing a slight increase with the increase in the number of layers. The main reason is that as the number of core layers increases, the stiffness of the core structure decreases, resulting in a greater capacity for elastic deformation. When the number of core layers increases from one to three, the yield stress, plateau stress, and specific energy absorption gradually decrease, while the densification strain is gradually increasing. From three to five layers, the densification strain and plateau stress remain essentially unchanged, while the yield stress and SEA slightly decrease with the increase in the number of layers. The SEA of the one-layer structure is 9.43 J/g. As the number of layers increases, the energy absorption capacity of the multi-layer structure tends to stabilize at an SEA of approximately 5.00 J/g, a decrease of 46.9%. The main reason is that the inter-layer nodal connection strength of the middle rings weakens with the increase of core layer numbers. In addition to vertical crushing deformation, the core rings undergo unstable torsional deformation at the contact points between adjacent core layers, which weakens the energy absorption capacity of the multi-layer ring lattice core.

### 6.2. Addition of Inner Panels

**Figure 30:**
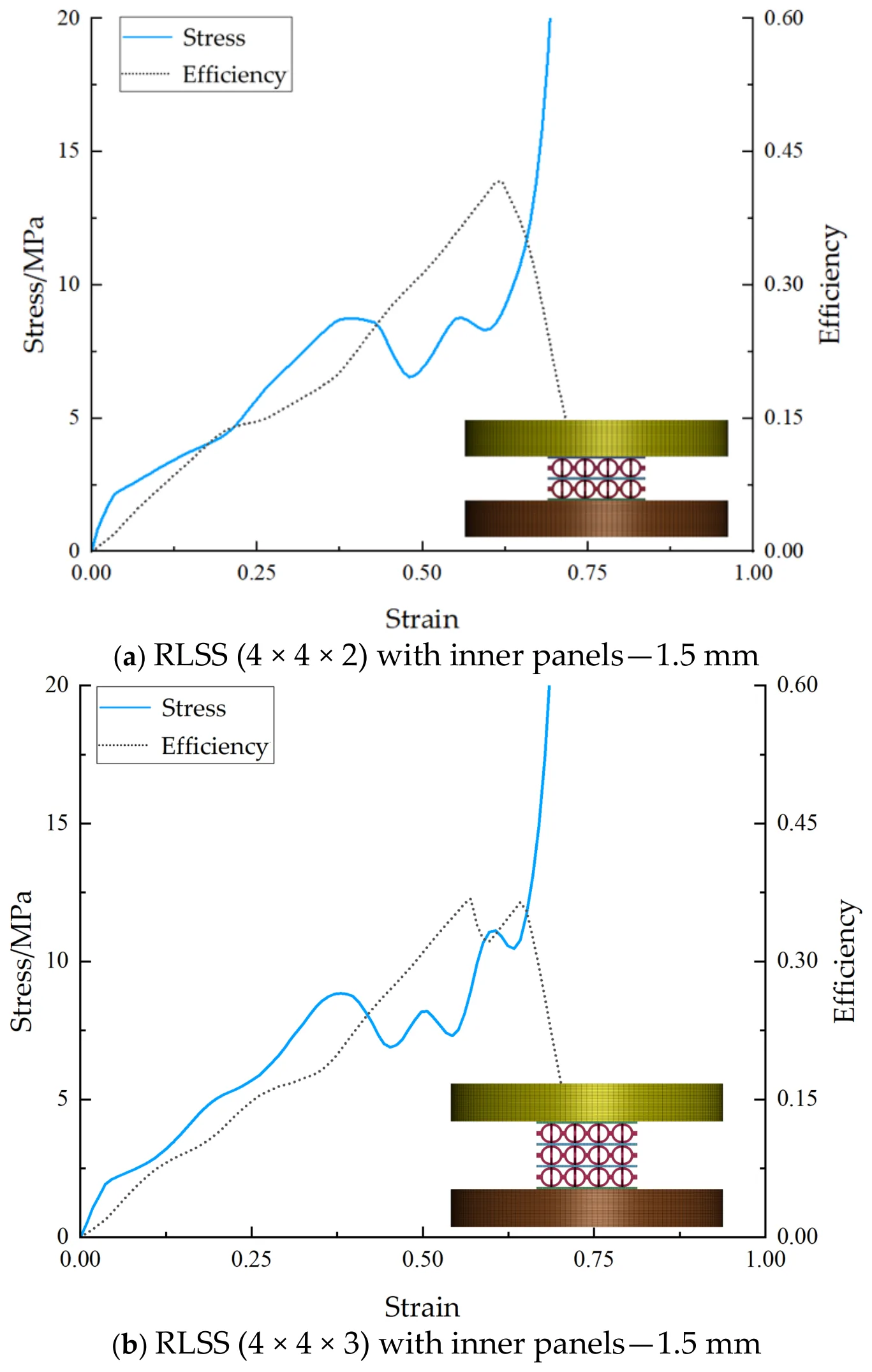
Energy absorption efficiency diagrams for two-layer and three-layer RLSSs with inner panels under quasi-static compression obtained from numerical simulation.

To overcome the weak inter-layer nodal connection strength of the lattice core in the RLSSs, numerical simulations of quasi-static compression were performed on two-layer and three-layer ring lattice sandwich structures with inner panels, which were 1.5-mm-thick SUS304 stainless steel plates. The obtained energy absorption efficiency diagrams and indicators are shown in Figure 30 and Table 6. Comparing Figure 20, Figure 29 and Figure 30, the stress–strain curve trend for the RLSSs with inner panels is essentially consistent with that of the one-layer RLSS. Further comparison of Table 3, Table 5 and Table 6 reveals that the yield strain, densification strain, yield stress, and plateau stress of the two-layer and three-layer structures with inner panels are in good agreement with those of the one-layer structure. Their energy absorption capacities are 2.05 and 3.24 times that of the one-layer structure, and 1.60 and 2.01 times that of the two-layer and three-layer structures without inner panels, respectively. The main reason is that the addition of inner panels strengthens the inter-layer nodal connection strength. The core constraint can be considered equivalent to that of the one-layer structure, and the crushing mode and energy absorption indicators of each individual core layer are approximately equivalent to those of the one-layer RLSS. It is worth noting that the specific energy absorptions of the two-layer and three-layer structures with inner panels are 48.8% and 43.8% of that of the one-layer structure, respectively. This is because the added inner panels increase the total mass of the lattice core, causing the SEA of the structure with inner panels to decrease as the number of layers increases.

**Figure 31:**
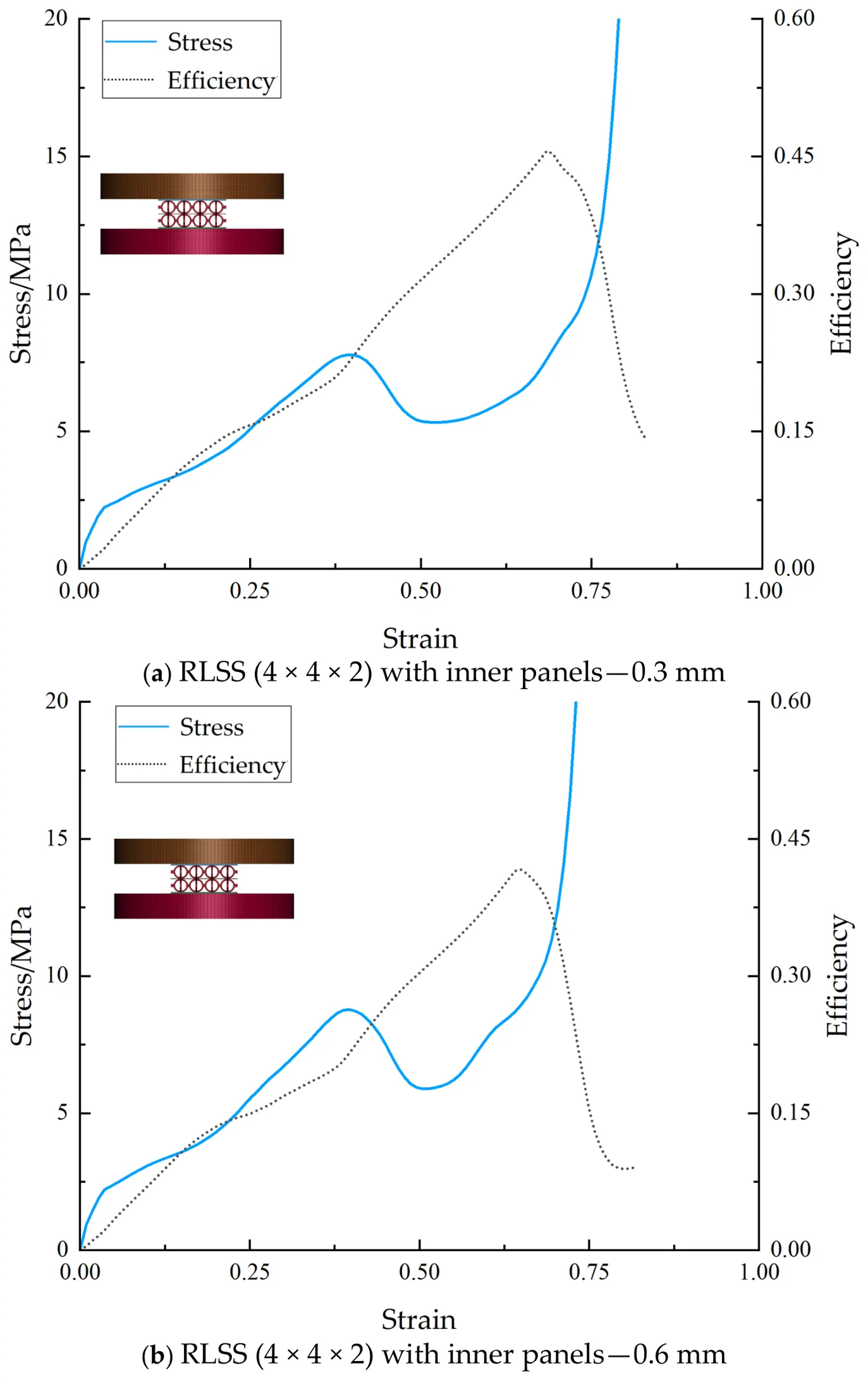

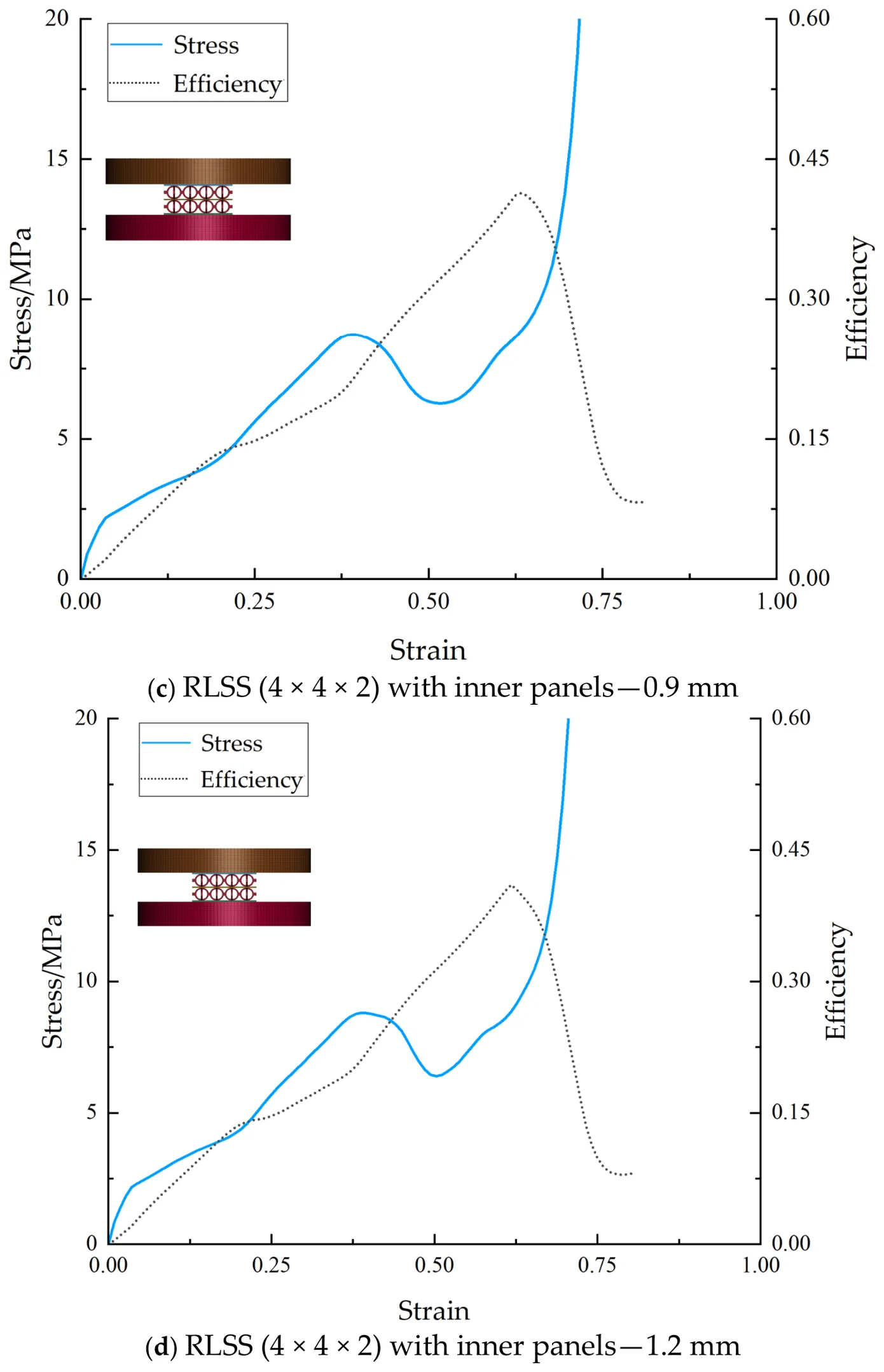
Energy absorption efficiency diagrams for RLSSs (4 × 4 × 2) with inner panels of varying thicknesses under quasi-static compression obtained from numerical simulation.

**Figure 32:**
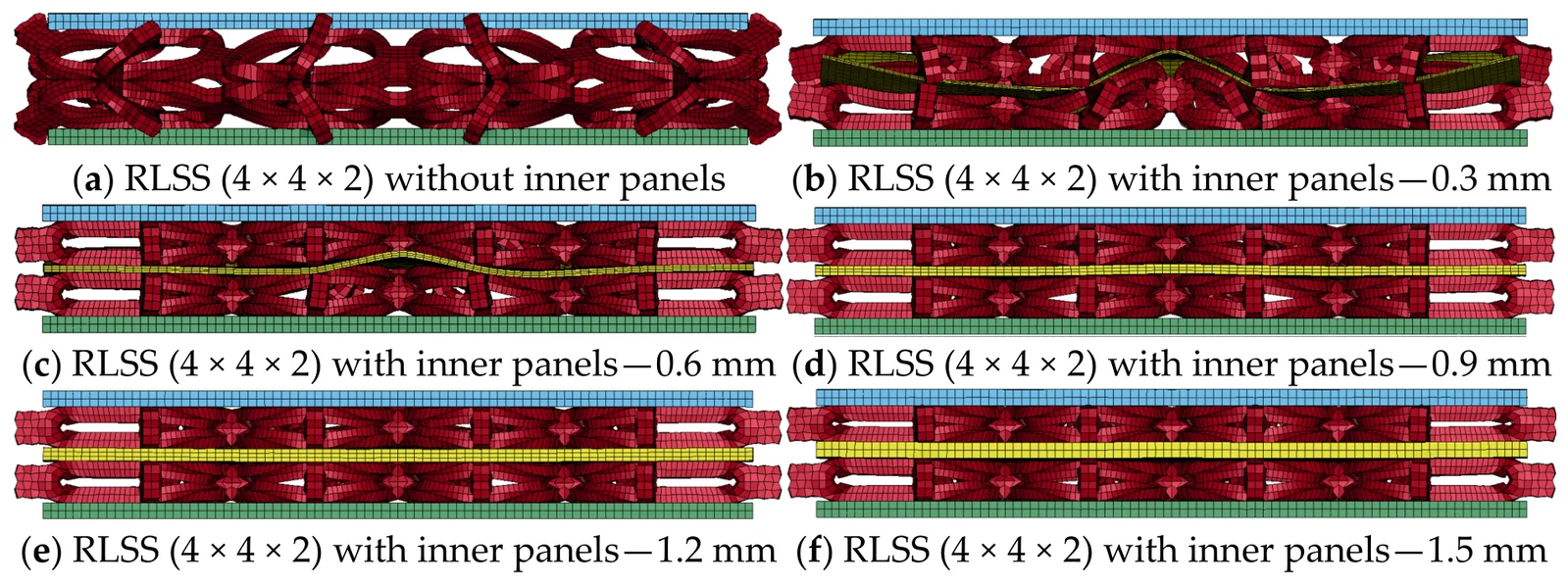
Deformation mode for RLSSs (4 × 4 × 2) with inner panels of varying thicknesses under quasi-static compression at densification strain obtained from numerical simulation.

To achieve the optimal inner panel thickness, numerical simulations of quasi-static compression were conducted on RLSSs (4 × 4 × 2) with inner panel thicknesses ranging from 0.3 mm to 1.2 mm. The obtained energy absorption efficiency diagrams and indicators are shown in Figure 31 and Table 7. Comparing Figure 20, Figure 30 and Figure 31, the stress–strain curves for the RLSSs with inner panels of varying thicknesses exhibit similar trends, which are basically consistent with those of the one-layer RLSS. The deformation of RLSSs (4 × 4 × 2) with different thicknesses of inner panels when reaching the densification strain is shown in Figure 32. From Figure 32, it can be observed that after adding inner panels between the core layers, the unstable torsional deformation phenomenon of the interlayer nodes significantly improves with the increase in interlayer thickness. When the interlayer thickness is 0.3 mm and 0.6 mm, the inner panel stiffness is relatively low, and the inner panel exhibits significant buckling deformation during the compression process of the core, resulting in a certain degree of torsional deformation in the core. When the inner panel thickness reaches 0.9 mm, the deformation of the RLSS (4 × 4 × 2) inner panel is almost negligible, and the crushing mode of the RLSS (4 × 4 × 2) with the inner panel is basically consistent with that of the RLSS (4 × 4 × 1). From Table 7, it can be observed that as the inner panel thickness increases, the yield stress of the structure slightly decreases, approximately equal to that of the one-layer RLSS, while the plateau stress and absorbed energy gradually increase, and the SEA gradually decreases. Analyzing Table 5 and Table 7, it can be found that when the inner panel thickness is 0.3 mm and 0.6 mm, the SEA of RLSS (4 × 4 × 2) is 7.19 J/g and 6.34 J/g, respectively, which is greater than that of the RLSS (4 × 4 × 2) without an inner panel (6.03 J/g), verifying the effectiveness of adding inner panels in enhancing the inner panel node connection while improving SEA.

Therefore, in practical engineering design, the choice of inner panel thickness should be guided by the specific application requirements: if weight efficiency is the primary concern (e.g., in aerospace or portable protective systems), a thinner inner panel (0.3 mm) is preferable as it offers the highest SEA; if the structure is weight-insensitive but space-constrained (e.g., in fixed energy infrastructure cladding), a thicker inner panel may be adopted to maximize the total energy absorption capacity. This trade-off should be carefully balanced according to the performance priorities of the target application.

### 6.3. Strain Rate

**Figure 33:**
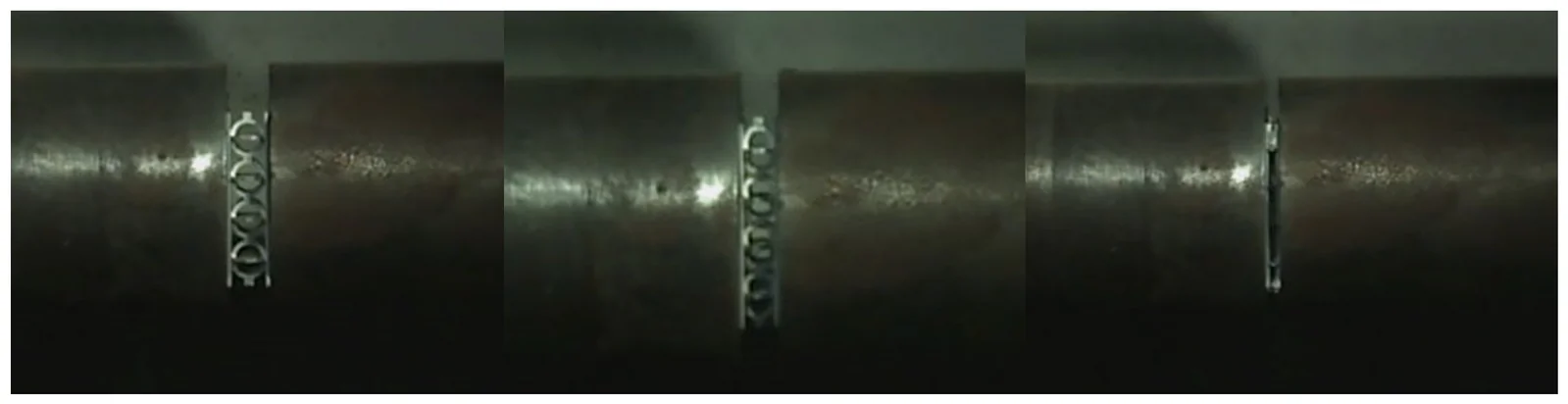
Deformation modes of the RLSSs in SHPB tests.

**Figure 34:**
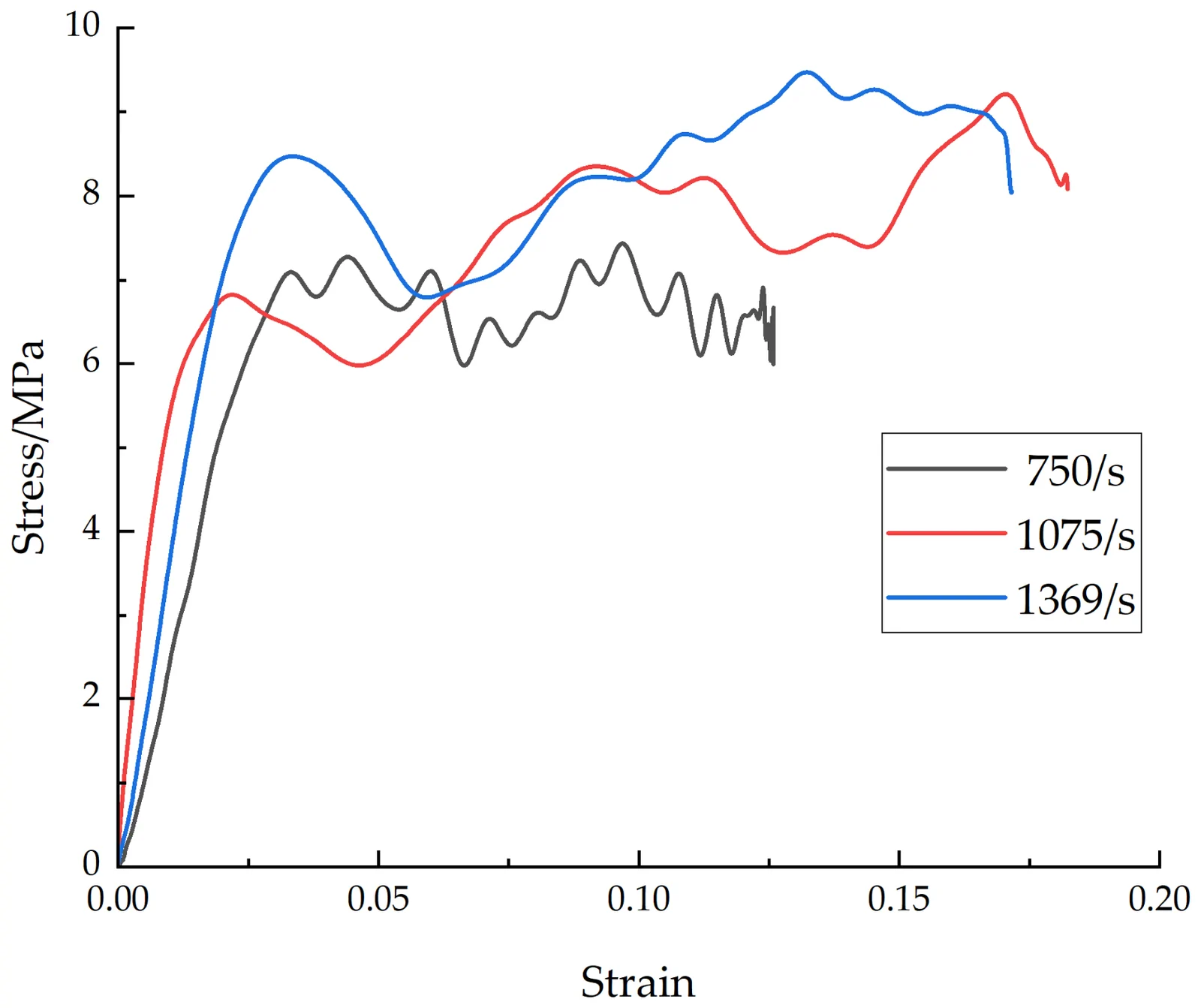
Stress–strain curves of RLSS (4 × 4 × 1) obtained from SHPB test.

SHPB impact tests were conducted on one-layer RLSSs at gas chamber pressures of 0.2 MPa, 0.4 MPa, and 0.6 MPa, with measured striker impact velocities of 12 m/s, 17.2 m/s, and 21.9 m/s, corresponding to strain rates of 750 s^−1^, 1075 s^−1^, and 1369 s^−1^, respectively. The dynamic deformation process of the one-layer RLSSs during the SHPB tests is shown in Figure 33. Comparing with Figure 5, it can be seen that the deformation mode of the RLSS under impact loading is essentially consistent with that under quasi-static compression. The stress–strain curves of the one-layer RLSSs at high strain rates are presented in Figure 34. Owing to the relatively large height of the specimens, complete high-strain-rate stress–strain curves could not be obtained. Therefore, only the plateau stresses of the one-layer RLSSs were calculated based on the available curves, and the results are listed in Table 8. It can be observed from Table 8 that, even though the dynamic impact load on the ring lattice sandwich structure is considerably underestimated, the dynamic mechanical properties under impact loading are significantly enhanced compared with those under quasi-static crushing, exhibiting a pronounced strain-rate effect, which indicates that the structure has great potential for applications in impact and blast protection.

### 6.4. Structural Configuration

**Figure 35:**
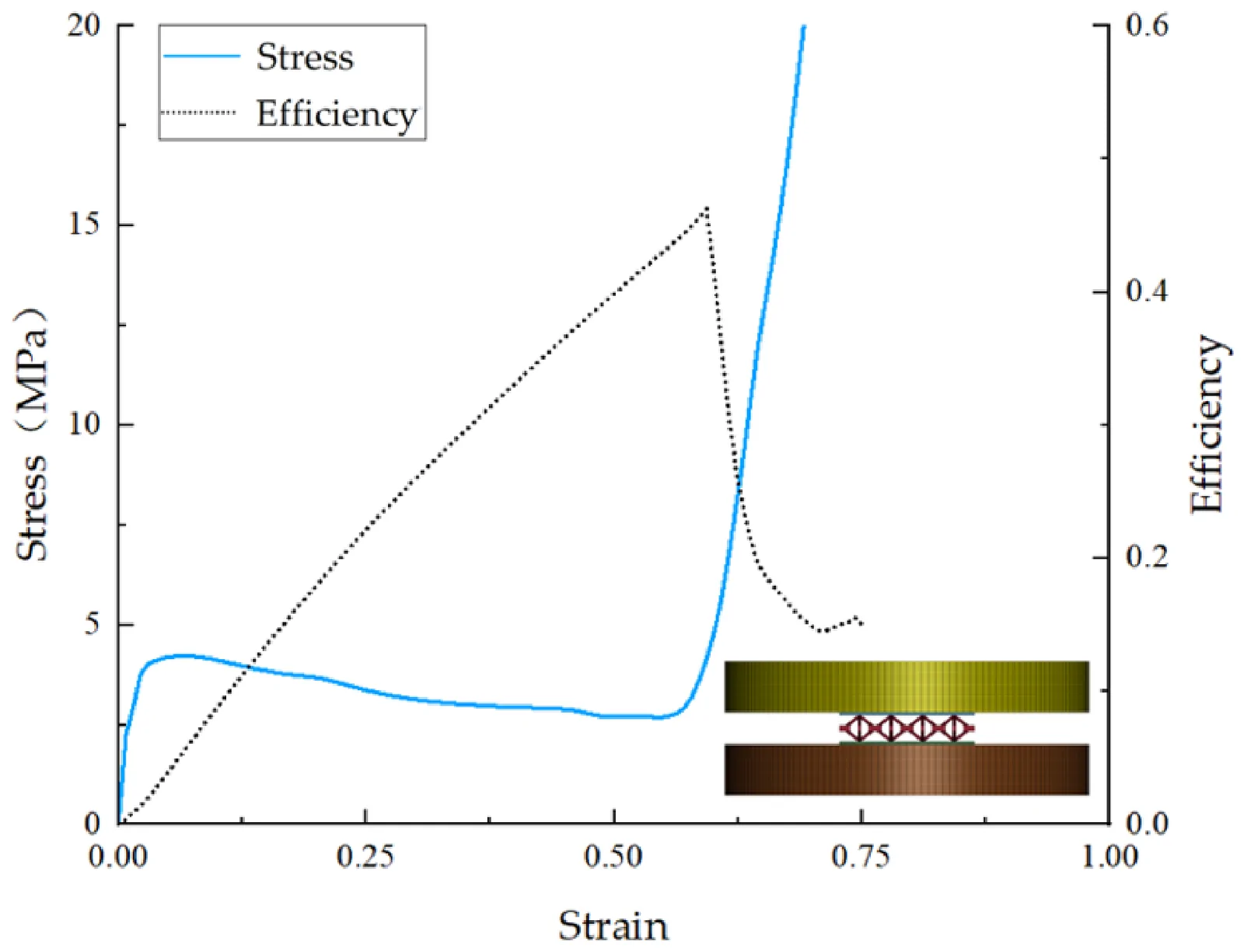
Energy absorption efficiency diagram for PLSSs (4 × 4 × 1) under quasi-static compression obtained from numerical simulation.

The energy absorption efficiency diagrams and indicators obtained from numerical simulations for the RLSS (4 × 4 × 1) and PLSS (4 × 4 × 1) are shown in Figure 20 and Figure 35, and Table 9. It can be seen from Figure 20 and Figure 35 that the stress–strain curves of the RLSS (4 × 4 × 1) and PLSS (4 × 4 × 1) are significantly different. After yielding under compression, the RLSS (4 × 4 × 1) enters a plateau stage with a strain-hardening characteristic, then increases sharply after reaching the densification strain. In contrast, the PLSS (4 × 4 × 1) exhibits a peak stress at yielding, after which the stress gradually decreases with increasing strain until a sharp increase upon reaching the densification strain. As seen in Table 9, the yield strain, densification strain, and energy absorption efficiency of the two structures are relatively close. The yield stresses of the RLSS (4 × 4 × 1) and PLSS (4 × 4 × 1) are 2.18 MPa and 4.03 MPa, respectively, with the former being only 54.1% of the latter. However, the plateau stresses of the one-layer RLSS (4 × 4 × 1) and PLSS (4 × 4 × 1) are 5.91 MPa and 3.32 MPa, respectively, with the former being 1.78 times the latter, reflecting the different trends in their stress–strain curves. The energy absorption (EA) and specific energy absorption (SEA) of the RLSS (4 × 4 × 1) are 224.45 J and 9.43 J/g, respectively, while those of the PLSS (4 × 4 × 1) are 114.21 J and 4.76 J/g. The values for RLSS (4 × 4 × 1) are 1.97 and 1.98 times those of the PLSS (4 × 4 × 1), indicating that the one-layer ring lattice structure possesses superior energy absorption capacity.

**Figure 36:**
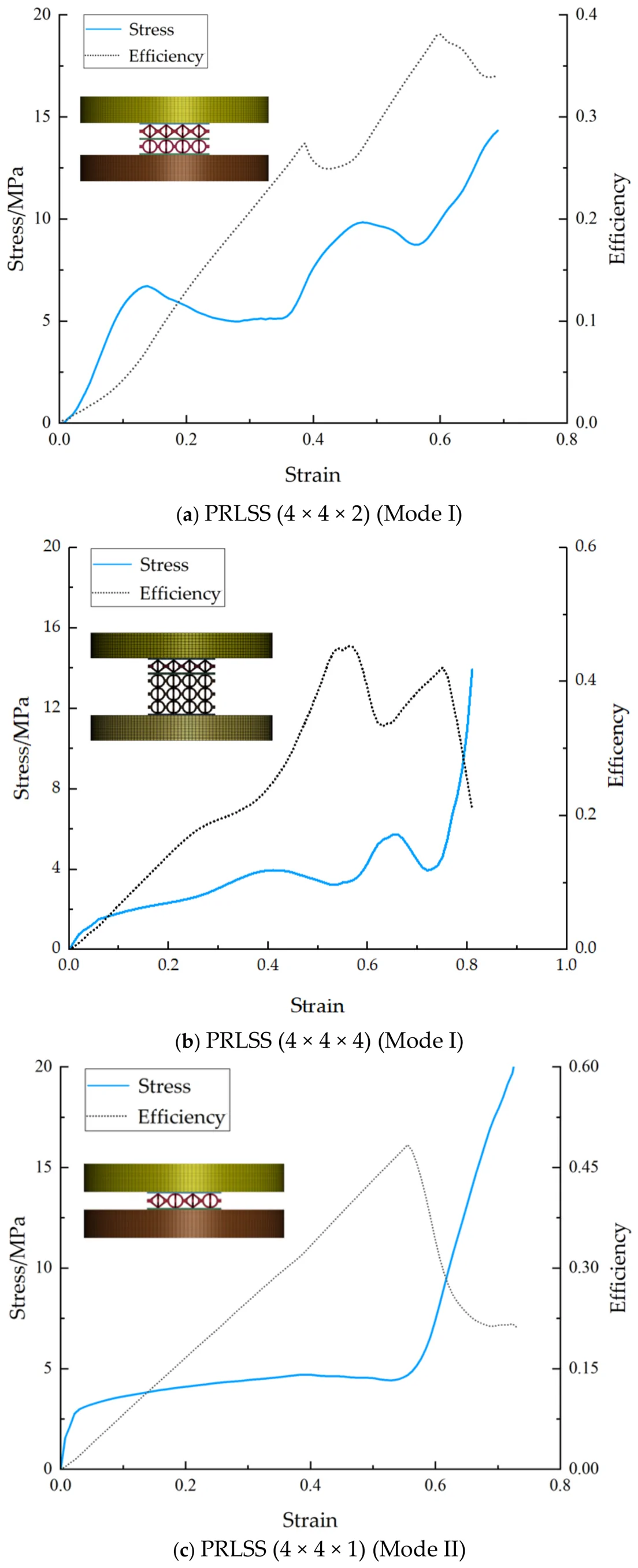
Energy absorption efficiency diagram of the lattice structure with hybrid core obtained from numerical simulation under quasi-static compression.

To further explore the architectural effects, two additional hybrid core configurations were examined (Figure 36). In Mode I (ring–pyramid–layer with inner panel), the number of pyramidal layers was held constant while the ring-core layer count was progressively increased. Together with the previously introduced hybrid core PRLSS, this yielded three distinct PRLSS variants. Their energy absorption characteristics, obtained through numerical simulation, are summarized in Table 10. The results indicate that an increase in the ring-layer number substantially reduces both the plateau stress and the energy dissipation capacity, with the PRLSS (4 × 4 × 2) configuration exhibiting the most favorable performance among the Mode I variants. This deterioration is primarily attributed to the additional ring layers promoting earlier local buckling, which collectively lower the crushing resistance and the overall energy-absorption efficiency. In contrast, Mode II (alternating ring and pyramid layers, PRLSS (4 × 4 × 1)) shows the lowest plateau stress fluctuation (PSF = 0.09) among all studied configurations, closely approaching the ideal energy-absorption curve. These hybrid designs offer versatile routes for either high total energy absorption (Mode I) or stable, predictable dissipation (Mode II). On the basis of the observed mechanisms, for Mode I designs aiming to enhance energy dissipation, it is advisable to limit the number of ring layers and to optimize geometric parameters, so as to delay buckling and enhance core-to-core interaction.

### 6.5. Novelty and Contribution

(1) A novel ring lattice sandwich structure (RLSS) is proposed and fabricated via an interlocking-assembly-brazing method. Unlike additive manufacturing, which is costly and time-consuming, this approach is economical, rapid, and suitable for large-scale production, thus facilitating practical engineering applications.
(2) For the first time, theoretical models for the dimensionless relative density and initial yield stress of the RLSS are established. The predicted values show good agreement with experimental results, with relative errors of 7.1% and 6.6%, respectively, providing a reliable design tool for this new topology.
(3) Systematic numerical simulations reveal key factors affecting the energy absorption of the RLSS, including the number of core layers, inner panel thickness, strain rate, and core configuration. For multi-layer structures, the inter-layer torsional instability is identified and effectively suppressed by adding thin inner panels (e.g., 0.3 mm), which enhances the specific energy absorption from 6.03 J/g to 7.19 J/g.

## 7. Conclusions

In this study, a ring lattice sandwich structure (RLSS) was designed and fabricated via the interlocking-assembly-brazing method. The mechanical behavior and energy absorption performance under compression were systematically investigated through experiments, numerical simulations, and theoretical analysis. The main conclusions are drawn as follows:

(1) Theoretical models for dimensionless relative density and initial yield stress of the RLSS are established. The predicted values agree well with experimental results, with relative errors of 7.1% and 6.6%, respectively, validating the theoretical framework.
(2) Quasi-static compression tests show that the one-layer RLSS exhibits an elastic stage, a strain-hardening plateau, and a densification stage, with a specific energy absorption (SEA) of 8.67 J/g. The two-layer structure, however, suffers from inter-layer torsional instability, leading to a lower SEA of 5.66 J/g.
(3) SHPB impact tests on the one-layer RLSS at strain rates of 750 s^−1^, 1075 s^−1^, and 1369 s^−1^ indicate a pronounced strain-rate effect, with dynamic increase factors of 1.14, 1.31, and 1.43, respectively, confirming the structure’s potential for impact and blast protection.
(4) Numerical simulations accurately reproduce the experimental crushing modes and load-displacement responses. For multi-layer RLSSs (2–5 layers), simulations reveal that weakened inter-layer nodal connections cause unstable torsional deformation, reducing SEA from 9.43 J/g (simulated one-layer) to approximately 5.00 J/g, a drop of 46.9%.
(5) Numerical parametric studies demonstrate that adding thin inner panels (e.g., 0.3-mm-thick SUS304 plates) effectively suppresses the torsional instability. A two-layer structure with a 0.3 mm inner panel achieves an SEA of 7.19 J/g, exceeding that of the two-layer structure without inner panels (6.03 J/g), while its crushing mode becomes nearly identical to that of the one-layer RLSS.
(6) Comparative simulations between the one-layer RLSS and an equivalent pyramidal lattice sandwich structure (PLSS) show that the RLSS has an SEA of 9.43 J/g, which is 1.98 times that of the PLSS (4.76 J/g). Hybrid core designs offer versatile energy-dissipation strategies: Mode I (ring–pyramid with inner panel) enhances total energy absorption through complementary layer interaction, with the optimal performance achieved when the ring-layer count is kept low to delay local buckling; Mode II (alternating ring and pyramid layers) achieves the lowest plateau stress fluctuation (PSF = 0.09) among all studied configurations, closely approaching an ideal energy-absorption curve with superior crushing stability.

In summary, the proposed ring lattice sandwich structure (RLSS), particularly when optimized with thin inner panels or hybrid configurations, demonstrates excellent potential for use as protective cladding against UAV-induced impact and blast threats. Future work will extend the present findings to blast loading conditions, further investigate the microscopic failure mechanisms and inter-ring interaction effects through combined experimental and numerical approaches.

## Figures and Tables

**Table 1:** Quasi-static mechanical parameters of the furnace-accompanied base material.

Elastic Modulus /GPa	Tangent Modulus /GPa	Yield Strain	Ultimate Strain	Yield Stress /MPa	Ultimate Stress /MPa
257.73	1.80	9.36 × 10^−4^	0.46	241.21	1072.85

**Table 2:** Results of mesh convergence analysis.

Mesh Size /mm	Number of Elements	Number of Nodes	$\sigma_{pl}$/MPa	Difference /(%)	EA/J	Difference /(%)
10.0	319,616	425,890	5.35		169.42	
5.0	39,952	67,748	6.04	11.4%	224.45	24.5%
2.5	11,922	26,608	5.89	−2.5%	215.30	−4.3%

**Table 3:** Energy absorption indicators of one-layer RLSS, two-layer RLSS, and hybrid core PRLSS from tests and simulations.

Type	$\varepsilon_{cr}$	$\varepsilon_{cd}$	$\sigma_{cr}$ /MPa	$\sigma_{pl}$ /MPa	E	PSF	EA/J	SEA /(J/g)
RLSS (4 × 4 × 1)—Test	0.04	0.63	2.24	5.91	0.46	0.27	206.39	8.67
RLSS (4 × 4 × 1)—Simulation	0.03	0.66	2.18	6.04	0.45	0.30	224.45	9.43
RLSS (4 × 4 × 2)—Test	0.04	0.66	1.85	3.66	0.51	0.16	269.28	5.66
RLSS (4 × 4 × 2)—Simulation	0.03	0.68	1.75	3.80	0.47	0.22	287.29	6.04
PRLSS—Test	0.05	0.72	1.11	3.40	0.41	0.21	434.70	1.55
PRLSS—Simulation	0.06	0.71	1.84	3.41	0.41	0.17	429.00	1.53

**Table 4:** Energy absorption portion of components in one-layer RLSS, two-layer RLSS, and hybrid core PRLSS from simulations.

Type	Internal Energy of Lattice Core/J	Portion	Internal Energy of Panels/J	Portion	Friction Energy/J	Portion
RLSS (4 × 4 × 1)—Simulation	196.1	87.4%	1.1	0.5%	6.9	3.1%
RLSS (4 × 4 × 2)—Simulation	247.3	86.1%	2.1	0.9%	22.6	9.7%
PRLSS—Simulation	383.9	89.5%	6.4	1.5%	29.4	6.9%

**Table 5:** Energy absorption indicators of RLSSs with different layers.

Type	$\varepsilon_{cr}$	$\varepsilon_{cd}$	$\sigma_{cr}$ /MPa	$\sigma_{pl}$ /MPa	E	PSF	EA/J	SEA /(J/g)
RLSS (4 × 4 × 1)—Simulation	0.03	0.66	2.18	6.04	0.45	0.30	224.45	9.43
RLSS (4 × 4 × 2)—Simulation	0.03	0.68	1.75	3.80	0.47	0.22	287.29	6.03
RLSS (4 × 4 × 3)—Simulation	0.04	0.73	1.47	2.95	0.54	0.22	361.74	5.06
RLSS (4 × 4 × 4)—Simulation	0.04	0.72	1.24	2.96	0.48	0.30	474.77	4.99
RLSS (4 × 4 × 5)—Simulation	0.05	0.72	1.17	2.93	0.45	0.37	579.76	4.87

**Table 6:** Energy absorption indicators of RLSSs with inner panels.

Type	$\varepsilon_{cr}$	$\varepsilon_{cd}$	$\sigma_{cr}$ /MPa	$\sigma_{pl}$ /MPa	E	PSF	EA/J	SEA /(J/g)
RLSS (4 × 4 × 2) with inner panels—1.5 mm	0.03	0.62	2.16	6.24	0.42	0.31	459.63	4.60
RLSS (4 × 4 × 3) with inner panels—1.5 mm	0.04	0.63	2.07	6.30	0.36	0.31	727.59	4.13

**Table 7:** Energy absorption indicators of RLSSs with inner panels of varying thicknesses.

Type	$\varepsilon_{cr}$	$\varepsilon_{cd}$	$\sigma_{cr}$ /MPa	$\sigma_{pl}$ /MPa	E	PSF	EA/J	SEA /(J/g)
RLSS (4 × 4 × 2) with inner panels—0.3 mm	0.04	0.68	2.24	5.45	0.46	0.23	417.88	7.19
RLSS (4 × 4 × 2) with inner panels—0.6 mm	0.04	0.64	2.21	6.02	0.42	0.28	434.94	6.34
RLSS (4 × 4 × 2) with inner panels—0.9 mm	0.04	0.63	2.19	6.13	0.41	0.30	442.64	5.60
RLSS (4 × 4 × 2) with inner panels—1.2 mm	0.04	0.63	2.17	6.18	0.40	0.30	452.61	5.05
RLSS (4 × 4 × 2) with inner panels—1.5 mm	0.03	0.62	2.16	6.24	0.42	0.31	459.63	4.60

**Table 8:** SHPB test parameters for the one-layer RLSS.

Strain Rate/(/s)	2.6 × 10^−3^	750	1075	1369
Plateau stress/MPa	5.91	6.75	7.76	8.43
Dynamic increase factor	/	1.14	1.31	1.43

**Table 9:** Energy absorption indicators of one-layer RLSS (4 × 4 × 1) and PLSS (4 × 4 × 1).

Type	$\varepsilon_{cr}$	$\varepsilon_{cd}$	$\sigma_{cr}$ /MPa	$\sigma_{pl}$ /MPa	E	PSF	EA/J	SEA /(J/g)
RLSS (4 × 4 × 1)—Simulation	0.03	0.66	2.18	6.04	0.45	0.30	224.45	9.43
PLSS (4 × 4 × 1)—Simulation	0.03	0.59	4.03	3.32	0.46	0.14	114.21	4.76

**Table 10:** Energy absorption indicators of PRLSSs.

Type	$\varepsilon_{cr}$	$\varepsilon_{cd}$	$\sigma_{cr}$ /MPa	$\sigma_{pl}$ /MPa	E	PSF	EA/J	SEA /(J/g)
PRLSS (4 × 4 × 2) (Mode I)—Simulation	0.13	0.60	6.68	7.15	0.38	0.25	468.85	4.68
PRLSS (4 × 4 × 3) (Mode I)—Simulation	0.06	0.71	1.84	3.41	0.41	0.17	429.00	1.53
PRLSS (4 × 4 × 4) (Mode I)—Simulation	0.06	0.57	1.52	2.96	0.45	0.19	343.57	1.36
PRLSS (4 × 4 × 1) (Mode II)—Simulation	0.03	0.56	2.97	4.18	0.48	0.09	132.81	5.56

## Data Availability

The original contributions presented in the study are included in the article, further inquiries can be directed to the corresponding author.

## Abbreviations

The following abbreviations are used in this manuscript:

EA	Energy Absorption
PLSS	Pyramid Lattice Sandwich Structure
PRLSS	Pyramid–Ring Lattice Sandwich Structure
PSF	Plateau Stress Fluctuation
RLSS	Ring Lattice Sandwich Structure
SEA	Specific Energy Absorption
UAV	Unmanned Aerial Vehicle

## Appendix A. Initial Yield Stress of One-Layer RLSS

### Appendix A.1. Initial Yield Load of Edge Rings [30,31]

For a rigid-plastic ring with a radius of *R*_0_ and a thickness and width of *t* in its cross-section, under the action of a pair of concentrated forces in opposite directions, four plastic hinges are required to form a collapsed structure.

Considering the balance of a quarter-ring segment, when the rotation angle of the quarter-ring segment is *θ*, the length *L_AB_* can be expressed as:

(A1) $$L_{AB}=\sqrt{2}R_{0}\cos(\frac{\pi }{4}-\theta )$$

*R*_0_ represents the radius at the center layer of the circular ring, which can be expressed as:

(A2) $$R_{0}=R-t/2$$

where *R* represents the outer diameter of the circular ring, and *t* denotes the thickness of the circular ring’s cross-section.

The force and torque acting on the quarter-ring segment are equivalent to two forces with equal magnitude but opposite directions. The equilibrium condition requires that these two forces must act along the same line, which can be expressed as follows:

(A3) $$\frac{2M_{p}}{P}=L_{AB}$$

For a circular ring, its plastic bending moment *M_p_* can be expressed as:

(A4) $$M_{p}=\frac{bh^{2}}{4}\sigma_{0}$$

where *b* and *h* represent the width and height of the circular arc section, respectively, both of which are equal to *t*; *σ*_0_ denotes the yield stress of the material.

When *θ* = 0, by combining Equations (A1)–(A4), the initial yield load *P*_01_ is obtained:

(A5) $$P_{01}=\frac{4M_{p}}{R_{0}}=\frac{\sigma_{0}t^{3}}{R-t/2}$$

### Appendix A.2. Initial Yield Load of Middle Rings [32]

For the middle rings, their initial collapse load is approximately similar to that of a built-in semi-circular arch under the action of a concentrated force. Consider a built-in semicircular arch with a radius of *R*_0_, a section thickness and width both of *t*, and a plastic bending moment of *M_p_*. Neglecting the influence of axial force, under the inward concentrated load *P*, five plastic hinges are required to form a collapsed structure.

Considering the equilibrium of the semi-circular arch, the equivalent resultant force $P^{\prime }$ has an opposite direction, and the distances from the five plastic hinges A, B, C, D, and E to the line of action of the resultant force are equal. Thus, we have:

(A6) $$L_{AA^{\prime }}=L_{BB^{\prime }}=L_{CC^{\prime }}=L_{DD^{\prime }}=L_{EE^{\prime }}=\frac{R_{0}}{4+2\sqrt{2}}$$

and

(A7) $$\frac{M_{p}}{P^{\prime }}=L_{AA^{\prime }}$$

Based on the equilibrium relationship, the initial yield load *P*_02_ can be expressed as:

(A8) $$P_{02}=\sqrt{2}P^{\prime }$$

By combining Equations (A2)–(A4), and (A6)–(A8), the initial yield load *P*_02_ can be obtained:

(A9) $$P_{02}=\frac{4M_{p}}{R_{0}}(1+\sqrt{2})=(1+\sqrt{2})\frac{\sigma_{0}t^{3}}{R-t/2}$$

Comparing Formula (A9) with Formula (A5), it can be seen that under the same conditions, the initial yield load of the middle ring is 2.44 times that of the edge ring.

### Appendix A.3. Initial Yield Stress of RLSS

Taking RLSS (m × n × 1) as an example, the number of complete edge rings *N*_1_ and number of complete middle rings *N*_2_ can be expressed as:

(A10) $$N_{1}=m+n$$

(A11) $$N_{2}=\left(m-1\right)n+\left(n-1\right)m$$

For RLSS (m × n × 1), its initial yield load *P*_0_ can be expressed as:

(A12) $$P_{0}=N_{1}P_{01}+N_{2}P_{02}=\left[2(1+\sqrt{2})mn-\sqrt{2}(m+n)\right]\frac{\sigma_{0}t^{3}}{R-t/2}$$

In the formula, *P*_01_ and *P*_02_ represent the initial yield loads of the single edge ring and the single middle ring, respectively.

Taking the RLSS (4 × 4 × 1) quasi-static compression test as an example, the yield strength *σ*_0_ of the lattice structural material is 241.21 MPa, the outer diameter of the ring is 6.5 mm, and the thickness of the ring is 1.5 mm. The initial yield load obtained from the quasi-static compression test is 10.0 kN, and the theoretical yield load calculated according to Formula (A12) is 9.34 kN, with a relative error of 6.6%. The degree of agreement between theory and experiment is good, verifying the correctness of the theoretical analysis.

For RLSS (m × n × 1), the total lengths *L_m_* and *L_n_* in the meridional and latitudinal directions are, respectively:

(A13) $$L_{m}=2m(R+d)$$

(A14) $$L_{n}=2n(R+d)$$

where *R* represents the outer diameter of the ring, and *d* represents half of the length of the joint between the rings.

By combining Equations (A12) and (A13), the initial yield stress *σ_ini_* of RLSS (m × n × 1) can be obtained:

(A15) $$\sigma_{ini}=\frac{P_{0}}{L_{m}L_{n}}=\frac{\left[2(1+\sqrt{2})mn-\sqrt{2}(m+n)\right]\sigma_{0}t^{3}}{4mn(R-t/2){\left(R+d\right)}^{2}}$$

For RLSS (m × n × 1) with large m and n, Equation (A15) can be simplified as:

(A16) $$\sigma_{ini}=\frac{1+\sqrt{2}}{2}\frac{\sigma_{0}t^{3}}{(R-t/2){\left(R+d\right)}^{2}}$$

As can be seen from Equation (A16), the key parameters determining the initial yield stress of RLSS (m × n × 1) are the yield strength *σ*_0_ of the ring material, the outer diameter *R* of the ring, the thickness *t* of the ring, and the connection length *d* between rings. For the same material, the initial yield stress of the ring lattice structure increases with the increase of the ring thickness *t*, and decreases with the increase of the outer diameter *R* of the ring and the connection length *d* between rings.
